# Dissecting Major Signaling Pathways throughout the Development of Prostate Cancer

**DOI:** 10.1155/2013/920612

**Published:** 2013-04-29

**Authors:** Henrique B. da Silva, Eduardo P. Amaral, Eduardo L. Nolasco, Nathalia C. de Victo, Rodrigo Atique, Carina C. Jank, Valesca Anschau, Luiz F. Zerbini, Ricardo G. Correa

**Affiliations:** ^1^Departamento de Imunologia, Instituto de Ciências Biomédicas, Universidade de São Paulo, Avenida Prof. Lineu Prestes 1730, 05508-900 São Paulo, SP, Brazil; ^2^Departamento de Análises Clínicas e Toxicológicas, Faculdade de Ciências Farmacêuticas, Universidade de São Paulo, Avenida Prof. Lineu Prestes 580, 05508-000 São Paulo, SP, Brazil; ^3^Departamento de Genética e Biologia Evolutiva, Instituto de Biociências, Universidade de São Paulo, Rua do Matão 277, 05508-900 São Paulo, SP, Brazil; ^4^Instituto Israelita de Ensino e Pesquisa Albert Einstein, Avenida Albert Einstein 627/701, 05652-000 São Paulo, SP, Brazil; ^5^International Center for Genetic Engineering & Biotechnology (ICGEB), Cancer Genomics Group and Division of Medical Biochemistry, University of Cape Town, Cape Town 7925, South Africa; ^6^Sanford-Burnham Medical Research Institute, 10901 North Torrey Pines Road, La Jolla, CA 92037, USA

## Abstract

Prostate cancer (PCa) is one of the most common malignancies found in males. The development of PCa involves several mutations in prostate epithelial cells, usually linked to developmental changes, such as enhanced resistance to apoptotic death, constitutive proliferation, and, in some cases, to differentiation into an androgen deprivation-resistant phenotype, leading to the appearance of castration-resistant PCa (CRPCa), which leads to a poor prognosis in patients. In this review, we summarize recent findings concerning the main deregulations into signaling pathways that will lead to the development of PCa and/or CRPCa. Key mutations in some pathway molecules are often linked to a higher prevalence of PCa, by directly affecting the respective cascade and, in some cases, by deregulating a cross-talk node or junction along the pathways. We also discuss the possible environmental and nonenvironmental inducers for these mutations, as well as the potential therapeutic strategies targeting these signaling pathways. A better understanding of how some risk factors induce deregulation of these signaling pathways, as well as how these deregulated pathways affect the development of PCa and CRPCa, will further help in the development of new treatments and prevention strategies for this disease.

## 1. Introduction

The long-term ineffectiveness of current treatments for prostate cancer (PCa) has spurred an increasing interest in understanding the molecular mechanisms that underlie PCa tumorigenesis [1]. Currently, PCa is considered the most common nonmelanoma neoplasia among men [2–4]. According to the current trends in population growth, the incidence of PCa will exceed 1.7 million new cases by 2030 [5]. In the United States, nearly 2.8 million men are potentially living with this condition, and approximately 240,000 new cases were diagnosed in 2012 [3]. PCa predominately affects elderly men with higher incidence [6], and it is more prevalent in Western countries [7], where the average life expectation is over 75 years old. In developing countries like Brazil, PCa has recently surpassed the population incidence of breast cancer, and it has become the most common tumor malignancy, with approximately 50,000 new cases occurring per year [4, 5]. Yet, there is a considerable heterogeneity in the mortality rates and incidence among different countries, probably due to the variable penetrance of some risk factors such as age, race, genetics (family history), diet and environmental factors [8], and also behavioral factors, like frequent consumption of dairy products and meat [9], smoking, and sexual behavior [10].

Several agents such as diet, life habits, and exposure to chemical agents have been correlated with risk of PCa development [8]. For instance, a broad study performed by a PCa prevention trial group (Seattle, USA) has found high correlations between the intake of polyunsaturated fat and the development of aggressive PCa [11]. Corroborating this study, a strong correlation has been found (over 50%) between obesity and aggressive PCa development in both African and Caucasian men [12]. In Brazil, for instance, PCa is more frequently related to higher socioeconomic classes [13]. The increase in animal fat consumption and reduction in fiber consumption, along with sedentarism, have been suggested to be related to higher risks of PCa progression, along other types for cancers [14]. Thus, fat consumption appears to be a major risk factor for PCa. The association between pesticide exposure and hormone-related cancers, such as PCa, has been extensively debated since the late 1990s [15]. On the other hand, several studies have inversely correlated mild exposure to sunlight to higher mortality or PCa incidence [16]. However, the exact factors responsible for a potential induction of PCa are still not fully understood.

The development of prostatic tumor in men is generally slow, taking up to 4 to 10 years to develop a 0.4 inch-size tumor [17]. PCa begins when the semen-secreting prostate gland cells mutate into tumor cells, proliferating at higher mitotic levels. Initially, the prostate cells begin to proliferate leading to tumor formation in the peripheral zone of the prostate gland. Over time these cancer cells eventually multiply to further invade nearby organs, such as the seminal vesicles, rectum, bladder and urethra [18]. During the initial metastatic stages, malignant cells from the primary tumor detach from their original site and migrate through blood and lymphatic vessels [19]. In the later stages, cancer cells eventually spread to more distal organs, including bones, liver, and lung [18].

PCa treatment has been conducted primarily by surgery and/or radiotherapy due to the intimate organ localization [4, 20]. A prostatectomy usually leads to an excellent prognosis with low risk of death from PCa after surgery [21]. However, deregulated production and secretion of growth factors by stromal cells within the PCa microenvironment, as well as mutations in androgen signaling pathway components and further physiological modifications, including angiogenesis, local migration, invasion, intravasation, circulation, and extravasation of the tumor, potentially lead to systemic recurrence of the cancer, including the appearance of focal tumor in advanced stage [22–26]. In this case, the preferred treatment is based on androgen-deprivation therapy (ADT), mostly including a luteinizing-hormone-releasing hormone (LHRH) [20]. In advanced PCa, ADT still remains the most effective therapy in initial stages, despite its temporary effectiveness (in general, between 18 and 24 months) [20, 27].

In order to study PCa, a variety of cell lines mimicking androgen-dependent and androgen-independent carcinogenic formations have been extensively used [28]. These cell lines have enabled researchers to directly test a series of antitumor drug candidates, such as tumor apoptosis inducers [29] or enhancers of antitumor immune response [30], as well as to evaluate the genomic foundations of PCa [31] and to further decipher the biological characteristics within cancer development [32, 33]. Alongside the *in vitro* studies, several animal models have been developed in order to confirm *in vitro* results by using a more clinically relevant approach [34, 35]. Mouse models for PCa can be obtained by systemic induction of gene mutations [36], xenografts [37], or by doxycycline-based inducible systems to overexpress certain target genes like in the case of AKT, which in turn induces tumorigenesis [38].

Many genetic alterations may be accountable for PCa induction, whereas mutations in genes responsible for the expression of proteins that participate in a variety of cell signaling processes can affect the decision of cell death or survival [39]. In this review, we will discuss the role of major cellular signaling pathways in the progression of PCa and some potential strategies to prevent this malignant outcome.

## 2. The Androgen Receptor Signaling Pathway in Prostate Cancer

### 2.1. Pathway Description

**Figure 1:**
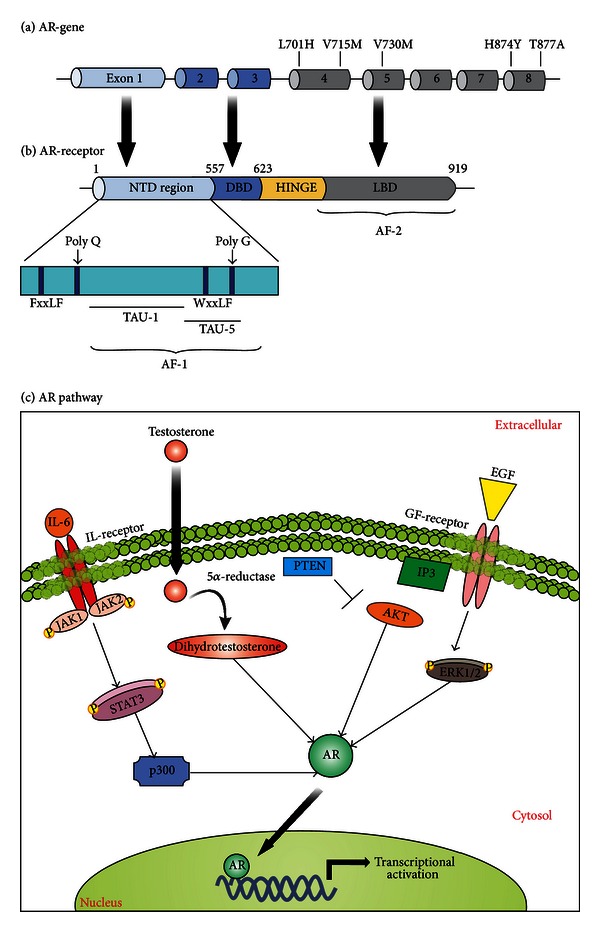
Androgen receptor (AR) signaling in prostate cancer. (a) Schematic representation of the AR gene, highlighting some major AR mutations and their exon localization. (b) Schematic representation of AR protein structure with indication of its functional domains. (c) AR-mediated signaling pathway. The androgen-receptor (AR) signaling pathway begins with the translocation of the testosterone to the cytoplasm, where it can be converted to dihydrotestosterone (DHT) and then promote the receptor dimerization and its further migration to the nucleus. A variety of signals, including PTEN-dependent downregulation, can also merge to AR stabilization and further activation (as indicated).

The androgen receptor (AR) signaling pathway promotes the differentiation of epithelial cells into male urogenital structures and encodes proteins that are necessary for the normal function of the prostate and for the initiation and maintenance of spermatogenesis [20, 40]. AR is a nuclear receptor that acts as a transcription factor [20], which is formed by four distinct functional domains like many other steroid-hormone receptors (Figure 1). The first region is composed of an N-terminal domain (NTD) that is constitutively active and has a transcriptional activation function (AF-1), executed by two transcriptional activation units (TAU-1 and TAU-2). The second region is a highly conserved DNA-binding domain (DBD), responsible for DNA binding specificity and for facilitating the dimerization and stabilization of the AR-DNA complex [20, 27]. The COOH-terminal ligand-binding domain (LBD) is another receptor site that is moderately conserved and equally important to mediate the binding to steroid hormones, which is the primary feature of the AR signaling pathway [20]. This site is also responsible for the direct binding between AR and the chaperone complex (Hsp90), which keeps the receptor in an inactive state but in a spatial conformation that allows affinity for androgens [41]. Upon binding to androgens, Hsp dissociates and releases AR from this complex, which further dimerizes and then translocates to the nucleus [27]. A fourth AR region contains the hinge region, a short amino-acid sequence that separates LBD from DBD and possesses a nuclear localization signal (NLS). This region is also important for the AR translocation to the nucleus through the interaction with the cytoskeletal protein Filamin-A (FlnA) [20], whose cytoplasmic localization is correlated with metastatic and hormone-refractory phenotype [20, 42].

### 2.2. Pathway Disruptions Associated with PCa and Therapeutic Targets

One of the major causes of CRPCa is AR overexpression, which can be related to gene amplification or transcriptional and/or translational upregulation and decreased degradation. AR gene amplification is observed in approximately 80% of the CRPCa cases, being the most common genetic alteration in this type of cancer [43]. However, gene amplification can only partially explain AR overexpression, and other mechanisms that promote this enhancement have been investigated [27]. AR regulates many genes through the binding of the AR-ligand complex to the DNA, specifically to androgen receptor binding sites (ARBSs) or androgen-responsive elements (AREs). These binding sites might be close to the target genes or acting as distal enhancers. During PCa progression, many androgen-regulated genes including UBE2C, CND1, p21, and p27 are up-regulated [43, 44]. In most of CRPCa conditions, where AR overexpression is found, prostate cells show more sensitivity to lower concentrations of the ligand [45].

AR mutations are rare in the initial phases of PCa, but they are very common in CRPCa [43]. These mutations might broaden AR specificity towards nonandrogenic molecules, or they can bypass the necessity of a ligand for proper transcriptional activity [27]. A considerable number of AR mutations have been characterized, showing that the promiscuous behavior of the receptor culminates in activation by adrenal androgens and other steroids hormones, including dehydroepiandrosterone (DHEA), progesterone, estrogens, and cortisol [27]. This phenomenon allows the prostatic epithelial cells to grow in an androgen-refractory way [40, 43, 46]. For this, there are three particular AR regions where mutations appear to give specific properties (Figure 1). The first region is between residues 701 and 730, and it enables resistance to adrenal androgens, glucorticoids and progesterone [27, 43], and mutations like L701H, V715M, and V730M are responsible for affecting these properties [27, 43]. In the second region, between residues 874–910, a T877A mutation has been described as the most frequent in CRPCa [43]. This alteration appears to affect the AR ligand specificity by changing the stereochemistry of the binding pocket, which expands the spectrum of ligands able to bind AR. This allows other hormones like DHEA, estrogen, progesterone, cortisone, and cortisol to activate AR [27, 40, 43]. Another mutation (H874Y) is also responsible for enhancing the transcription sensitivity of AR towards steroids like adrenal androgens or antiandrogens [43]. The third mutational site occurs between residues 670–678, located at the boundary of the hinge and LBD regions, that enhances the transactivation activity of AR in response to dihydrotestosterone (DHT). Other mutations in the amino terminus also occur but at a low frequency [27].

Transcription factors play a key role in AR expression and act positively or negatively in gene regulation. For instance, cAMP response element-binding proteins (CREB) have been reported to significantly increase during PCa progression, which ultimately enhances AR transcriptional levels [46]. The proto-oncogene Myc is well known to be involved in cancer formation [46] and it also participates in AR transcription, acting as a predictor of biochemical recurrence after radical prostatectomy (RP) [46, 47]. The member of the activator protein-1 (AP-1) c-Jun is known to suppress AR expression, but it also acts as a coactivator of this receptor [46, 47]. Another transcription factor that positively regulates AR transcription is FOXO3a, which binds to the Foxo-response element in the AR promoter region. The Lymphoid enhancer-binding factor 1 (LEF1) is a nuclear transducer that indicates a link between Wnt signaling and PCa, as Wnt1 leads to activation of LEF1 and it increases AR transcription [46]. Other transcription factors, like NF-*κ*B and Twist-1, have a positive correlation with AR expression, suggesting a key role in the progression and in the CRPCa state [46].

Another mechanism to bypass the requirement of ligands for AR activity is the presence of splice variants of AR transcripts. Alternative splicing events occur in approximately 90% of human genes and such events are evident in PCa [27, 43] where, in fact, it is an important mechanism of PCa resistance to AR-targeted therapy and further progression to CRPCa. Recent studies have identified several AR splice variants, and, despite having slightly different structures, a common characteristic is the absence of the LBD and the AF-2 domain in these isoforms [41]. The absence of LBD leads to loss of repression activity of this domain in the receptor, and potential hormone-independent AR activity [41]. It has been suggested that some AR variants may have an exclusive cytoplasmic function, although it has been demonstrated that truncated AR variants still show a nuclear localization that is enough to support transcriptional activity [41]. It has also been demonstrated that these AR variants can access the nucleus independently of the Hsp90 chaperone complex [41]. The clinical relevance of these variants is currently under investigation, and, due to the frequent identification of these splice variants in PCa metastases and CRPCa [27], these molecules could be envisioned as potential therapeutic targets.

Alterations of AR transcriptional activation induce deregulated proliferation and survival of prostate cells. For instance, it has been reported that androgens enhance the transcription of SENP1, a member of SUMO-specific protease family, showing that the regulation of AR signaling through this protease is based on a positive feedback mechanism [48]. Similarly, the regulation of the cell cycle regulator cyclin D1 by SENP1 contributes to cancer progression [49]. Therefore, SENP1 has emerged as an important prognostic marker and also a therapeutic target [38, 49]. Moreover, considering that the AR receptor is a phosphoprotein, changes to its phosphorylation profile would clearly have an impact on its function [20, 27, 40]. The use of pharmacological agents that modulate the AR posttranslational portfolio could be considered as an alternative approach for further interventions.

## 3. The NF-***κ***B Pathway in Prostate Cancer

### 3.1. Pathway Description

**Figure 2:**
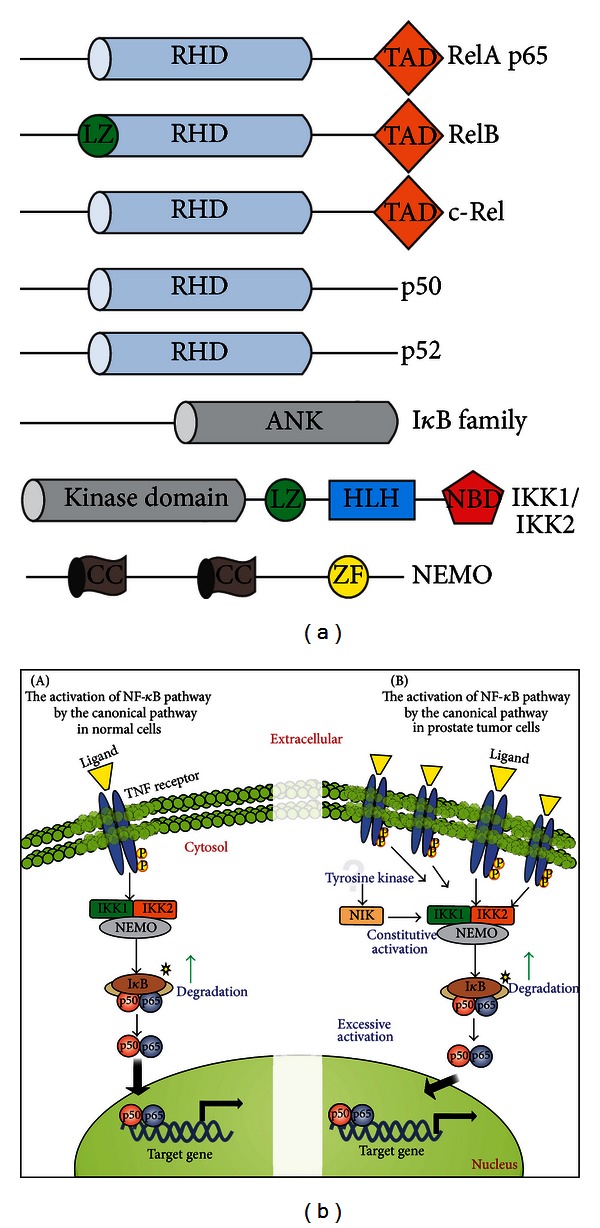
The NF-*κ*B signaling and prostate cancer. (a) Domain structure of NF-*κ*B family members and its direct modulators I*κ*B and IKK. The last two NF-*κ*B members p50 and p52 are derived from the C-terminal processing of p105 and p100, respectively. All NF-*κ*B family members contain an N-terminal Rel-homology domain (RHD) that governs the DNA binding, protein dimerization, and interaction to I*κ*B. The Rel subfamily, RelA, RelB, and c-Rel, also contain a C-terminal transcriptional activation domain (TAD) and the subunit RelB has an additional leucine zipper (LZ) domain at the N-terminus. The I*κ*B family mainly consists of I*κ*B*α*, I*κ*B*β*, I*κ*B*γ*, I*κ*B*ε*, and BCL-3 proteins (p100 also operates as an I*κ*B-like protein in the non-canonical pathway). The I*κ*B proteins contain ankyrin-repeat motifs (ANK) in their C-terminal region that interact with the RHD of NF-*κ*B proteins and then prevent their nuclear translocation and DNA binding. The I*κ*B kinase (IKK) complex is primarily composed of the two catalytic subunits IKK1 (or IKK*α*) and IKK2 (or IKK*β*) and the scaffolding protein NEMO (or IKK*γ*). IKK1 and IKK2 are structurally related and both contain an LZ domain and a helix-loop-helix region (HLH), with a C-terminal portion containing a NEMO binding domain (NBD). NEMO has an alpha helical region along with two coiled-coil (CC) regions and a putative zinc finger (ZF) domain. (b) The TNF-dependent NF-*κ*B signaling pathway. The canonical pathway in normal cells is used as an example for the signaling through TNF receptor. The activated IKK complex phosphorylates I*κ*B that is then degraded by the proteasome. Upon degradation of I*κ*B, the subunits of NF-*κ*B are released and the complex is free to migrate to the nucleus. The canonical NF-*κ*B pathway in prostate tumor cells is often constitutively activated, potentially due to increased levels of specific receptors like TNF receptors (TNFRs), which dramatically increase I*κ*B degradation and the translocation of NF-*κ*B dimers to the nucleus to activate *κ*B-responsive genes involved in the development and progression of the tumor. Additionally, undetermined tyrosine kinase subpathways lead to NIK activation, which induces constitutive IKK activity and then constitutive NF-*κ*B activation in androgen receptor-negative prostate cancer cell lines.

The nuclear factor kappa B (NF-*κ*B) signaling pathway is involved in a variety of physiopathological conditions, including inflammation, autoimmune disorders, and cancer. In humans, the NF-*κ*B family is composed of five members: p65 (RelA), p100/p52, p105/p50, c-Rel and RelB (Figure 2(a)). NF-*κ*B proteins form homo- or heterodimeric structures that, after activation, function as transcriptional factors through binding to *κ*B enhancer sites along the DNA. The canonical NF-*κ*B pathway involves the phosphorylation of the inhibitory I*κ*B proteins by the I*κ*B kinase complex (IKK) (composed of the catalytic subunits IKK*α* and IKK*β*, and the regulatory scaffolding protein NEMO), which results in the ubiquitination and further degradation of I*κ*B by the proteasome, thus releasing the NF-*κ*B dimers to translocate to the nucleus and activate *κ*B-responsive target genes. In contrast, a non-canonical NF-*κ*B pathway is detected in a more cell-specific fashion (including lymphoid tissue and immune-related cells), and it involves an IKK*α*-dependent p100 processing instead of the typical I*κ*B degradation. The non-canonical pathway is activated by specific stimuli that include Lymphotoxin-*β* (LT*β*) and B cell-activating factor (BAFF), whereas the canonical pathway is activated by a broader spectrum of stimuli, such as tumor necrosis factor *α* (TNF-*α*) and interleukin 1 (IL-1) and is often related to tumorigenesis, including leukemias, lymphomas, and some solid tumors [50–54]. Some NF-*κ*B target genes have important antiproliferative and apoptotic roles and may contribute to the development, progression, and resistance of certain tumor cells. 

### 3.2. Pathway Disruptions Associated with PCa and Therapeutic Targets

Molecular strategies that target NF-*κ*B have been shown to suppress prostate cancer, in terms of both prevention and further therapy [55–58]. For instance, the effect of specific IKK inhibitors in the growth and survival of androgen-dependent and independent PCa cell lines has been determined. The results indicate that, regardless of the AR status and androgen dependency, cell growth is remarkably affected [59]. Thus, the identification of NF-*κ*B responsive genes linked to PCa progression represents a critical step toward a better understanding and treatment of this disease. Some genetic alterations have been identified by the differential mRNA expression between tumor tissues versus normal tissues. For example, during androgen-independent tumorigenesis in the prostate, NF-*κ*B expression is elevated at both mRNA and protein level [60]. These studies indicate that the NF-*κ*B pathway can be constitutively activated in PCa, since an increased expression of interleukin 6 (IL-6) in androgen-independent PCa cell lines (PC-3 and DU145) was consistently observed. This deregulation of IL-6 expression in prostate cancer cells is in fact mostly mediated by the constitutive NF-*κ*B activation [61], and this activation occurs through signal transduction involving the upstream effectors NF-*κ*B inducing kinase (NIK) and IKK. Therefore, NF-*κ*B also targets a transcription regulatory element of the prostate-specific antigen PSA, which is an important marker for development and progression of PCa [62, 63]. 

The proinflammatory cytokine TNF-*α*, a prototypical NF-*κ*B inducer and also a downstream target gene, is highly expressed in PCa, and the TNF receptors TNFR1 and TNFR2 are also expressed at higher levels in the tumor epithelium when compared to normal prostate epithelium (Figure 2(b)) [64]. The levels of TNF-*α* in the serum are associated with the pathological data and the prognosis of PCa patients [65]. High expression of TNF-*α* has been correlated with increased survival and proliferation of PCa cells, angiogenesis, metastasis, and changes in the response to chemotherapeutic agents [66]. Experiments using PC-3 and DU145 cell lines treated with psoralidin (TNF-*α* inhibitor) indicate that this cytokine could be one potential therapeutic target. TNF-*α* inhibition by psoralidin inhibits NF-*κ*B via p65 and other upstream molecules, including the survival protein families IAPs (inhibitor of apoptosis proteins) [67]. The IAP proteins inhibit two major pathways that normally initiate the activation of the cysteine protease caspases, the mitochondrial (intrinsic) and the death receptor (extrinsic) pathways. The combined inhibition of IAPs and TNF-*α* could be attractive for PCa therapy, since IAPs modulate apoptotic events and TNF-*α* affects cell survival and proliferation via NF-*κ*B [68].

Recent clinical data and *in vitro* studies have suggested that NF-*κ*B directly interferes with AR signaling. NF-*κ*B is associated with increased AR expression and higher binding activity in androgen-independent xenografts [69]. In fact, AR has been described as a NF-*κ*B target gene, whereas p65/RelA activity causes an increase of AR at both mRNA and protein levels [70]. Moreover, endogenous AR expression can be induced by p65 in human prostate cancer cells, and this induction is associated with increased expression of downstream AR targets and enhanced growth and/or survival of prostate cancer cells [70]. Complex formation including the non-canonical p52 and AR has also been described, where it causes an increase in nuclear localization and binding of AR to DNA even in the absence of its ligand. This ligand-independent AR activation has similarities to the non-canonical NF-*κ*B signaling, since both pathways depend on IKK1 activity to phosphorylate the p100 precursor and by STAT3 phosphorylation [71]. NF-*κ*B and STAT3 share a subset of target genes during tumorigenesis, including PAI-1, Bcl-3, Bcl-2, and GADD45*β*. For this, the cooperation between STAT3 and NF-*κ*B pathways is required [72], in such a way that NF-*κ*B members physically interact with STAT3. This interaction can result in a synergy of specific gene transcription or repression regulated by NF-*κ*B/STAT3. It has been suggested that nonphosphorylated STAT3 can bind to the NF-*κ*B complex, thus facilitating its activation independently of IKK activity, supporting the idea that STAT3 may prolong the presence of active NF-*κ*B dimers in the nucleus. Thus, STAT3 may represent an important mechanism that ensures continuous NF-*κ*B activation in cancer cells [72].

The regulation of NF-*κ*B by the tumor suppressor gene p53 has also been observed in many types of hematopoietic and solid tumors [73]. The interaction between p53 and NF-*κ*B reveals that, despite its role as a tumor suppressor, NF-*κ*B becomes activated after reactivation of p53 even when the p53-induced apoptosis requires the participation of NF-*κ*B. Thus, activation of NF-*κ*B in apoptosis is additionally related to a hyperactivation of p53 [73]. Because NF-*κ*B and p53 can be eventually activated by the same stimuli, the balance of their activities is crucial for cell fate decision. An important mechanism of communication between these two pathways is the binding competition for CBP and p300, which are necessary for the selective activation of these factors [74]. 

## 4. The PI3K/AKT Pathway in Prostate Cancer

### 4.1. Pathway Description

The Phosphoinositide 3-kinase/AKT (PI3K/AKT) pathway is a key signal transduction pathway that links multiple classes of membrane receptors to many essential cellular functions, such as cell survival, proliferation, and differentiation [75–77]. PI3K molecules are divided into three major classes: class I (IA and IB) molecules, which have one catalytic and one regulatory subunit and can bind to receptor tyrosine kinases, G-protein coupled receptors and oncogenic proteins, such as small G protein RAS, to transduce their signals, and class II and III molecules which have a single catalytic subunit and can bind to several receptors, such as RTKs or cytokine receptors (class III molecules have been shown as important mediators of signaling through the mammalian target of rapamycin, mTOR). After activation of PI3K, these molecules can induce recruitment and activation of the serine/threonine-specific protein kinase AKT (also called protein kinase B, PKB) through phosphorylation-induced activation of transmembrane phosphatidylinositol (4,5) bisphosphate (PIP2) into phosphatidylinositol (3,4,5) trisphosphate (PIP3). PIP3 can recruit AKT through its pleckstrin homology domain [78, 79], a conserved protein module identified in many proteins involved in cell signaling or as cytoskeleton constituents. Activated AKT can subsequently phosphorylate and activate several other proteins, such as mTOR, glycogen synthase kinase 3, and FOXO members (the forkhead box family of transcription factors). Ultimately, AKT's action induces and regulates a large array of cellular processes [80, 81]. Considering that PI3K/AKT signaling is related to cell survival and proliferation, it is reasonable to link PI3K/AKT to cancer development.

### 4.2. Pathway Disruptions Associated with PCa and Therapeutic Targets

PI3K/AKT pathway is deregulated in the majority of solid tumors [82]. In PCa, it has been estimated that PI3K/AKT/mTOR signaling is up-regulated in 30%–50% of the cases, often due to the loss of PTEN function [83, 84], which leads to AKT hyperactivation. PTEN (phosphatase and tensin homolog) is responsible for the dephosphorylation of PIP3 to PIP2 and, in this way, negatively controls the activity of PI3K/AKT signaling. Interestingly, it is not clear whether or how direct mutations in AKT can lead to PCa [85]. PTEN is haploinsufficient in PCa, and its genetic dose is linked to PCa progression, in which total loss of function can be correlated with more advanced PCa, as seen in artificially created mouse models [86]. Complete PTEN inactivation in the prostate leads to a noninvasive PCa phenotype in mouse models, suggesting that other mutations might drive the appearance of more invasive tumors [87]. In fact, mutations in p53 or in the cyclin-dependent kinase inhibitor p27KIP1, when combined with loss of PTEN, have been linked to more aggressive PCa *in vivo* [87, 88]. Besides *PTEN* gene deletion, other mechanisms seem to contribute to loss of PTEN function. For instance, the action of microRNAs (miRNAs)—small, single-stranded RNA sequences which function as posttranscriptional regulators of gene expression—on PTEN inactivation has been recently described, with the characterization of miR-22 and miR-106b~25 as *PTEN*-targeting miRNAs aberrantly expressed in PCa [89]. It is also known that nuclear exclusion of PTEN is important for the development of tumors, including PCa [90]. In fact, it has been described that nuclear PTEN interacts with the anaphase-promoting complex (APC/C) and induces its association with CDH1 (APC/C activator protein), thereby enhancing the suppressive capacity of the APC-CDH1 complex to advance cell division [91], thus indicating a role for nuclear PTEN in PCa suppression.

The AKT hyperactivation induces high proliferative levels and resistance to apoptosis, an example of which is TRAIL resistance. TRAIL is a member of the tumor necrosis factor superfamily that specifically promotes apoptosis in cancer cells [92]. Indeed, treatment of PCa cells with the PI3K inhibitor LY294002 induces sensitization of these cells to TRAIL-induced apoptosis [93]. The excessive PI3K/AKT activation observed in PCa cells is accompanied by the presence of certain PI3K subunits that are not usually expressed in non-hematopoietic cells, such as p110*δ*. Augmented p110*δ* expression is correlated with inhibition of PTEN activity and further AKT activation [94]. Besides p110*δ*, transgenic mice with constitutive expression of p110*β* indicate that this molecule can be also linked to neoplasia formation [95].

**Figure 3:**
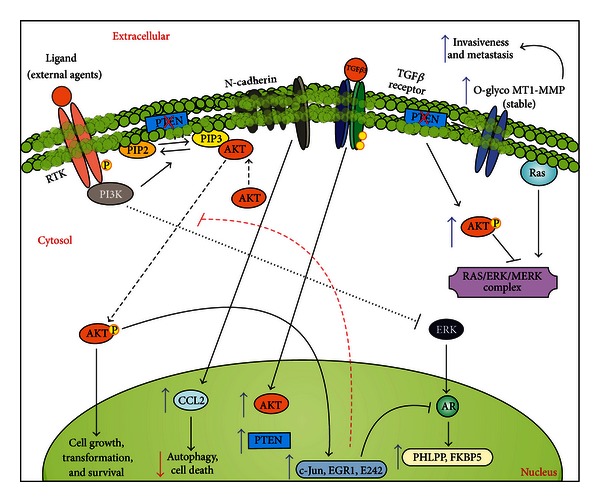
The PI3K/AKT signaling in prostate cancer. PI3K/AKT can induce enhanced activation of cancer cells by direct downstream effects. At the same time, this pathway (and downstream target genes) might affect the action of ERKs, which could lead to inhibition of AR-dependent activation, thus favoring an AR-independent growth. Conversely, AR pathway target genes can limit the PI3K/AKT pathway, favoring an AR-dependent tumor growth. A deregulated PI3K pathway (usually due to mutated or null PTEN) can also inhibit the Ras/MEK/ERK pathway, through enhanced activation of AKT. PI3K/AKT can also enhance the presence of stable metalloproteinase receptors (MT1-MMP), which favors invasive and metastatic phenotypes for these tumor cells. TGF*β* signaling (through TGF*β*3 ligation) can have a dual role in PI3K/AKT in PCa cells; for instance, benign cell lines enhance the expression of AKT and subsequent activation of this pathway following TGF*β*3 engagement; malignant cell lines enhance PTEN expression in response to TGF*β*3 engagement. Finally, N-cadherin enhanced expression in PCa cells leads to enhanced production of CCL2, which avoids autophagy in part through PI3K/AKT pathway.

PI3K/AKT pathway seems to act in conjunction with other proteins implicated in PCa cell growth. For example, AKT interacts with MST1, a hippo-like serine-threonine kinase [96]. Mst1 plays a critical role in the regulation of programmed cell death and it has been implicated in PCa development [96]. Interestingly, MST1 has been detected in AR-chromatin complexes, and forced expression of MST1 reduces AR binding to androgen-responsive elements along the PSA promoter [97]. MST1 also suppresses PCa cell growth *in vitro* and tumor growth *in vivo *[97]. AKT is able to phosphorylate a highly conserved residue Thr^120^ of MST1, which leads to inhibition of its kinase activity and nuclear translocation, as well as the autophosphorylation of Thr^183^ [98], having a positive role in PCa progression. Another example relates to a non-membrane tyrosine kinase called Acetate Kinase (Ack1) that is recruited by the upstream receptors and activates AKT through Tyr-176 phosphorylation, favoring the development of PCa [99]. Also, the polycomb group silencing protein Bmi1 can be phosphorylated by AKT, which enhances its oncogenic potential in PCa. Overexpression of Bmi1 can act in combination with PTEN haploinsufficiency to induce invasive carcinogenic formation in the prostate [100]. Recently, it was described that the deficiency of the Sprouty protein 2 (SPRY2) acts with the epidermal growth factor receptor (EGFR) system (RTK) and loss of PTEN to drive hyperactivation of PI3K/AKT via enhanced RTK trafficking in PCa [101]. It is also important to note that insulin-like growth factor (IGF) is an upstream effector on AKT signaling, and IGF up-regulation (which activates AKT) could promote the development of PCa *in vivo* [102, 103], suggesting an inter-relationship between IGF and AKT signaling in PCa. Finally, the Myc oncogene, a downstream target of PI3K/AKT pathway, commonly upregulated in many types of cancer [104], appears to act synergistically with AKT in the development of prostate tumorigenesis by altering, for instance, its sensitivity to mTOR inhibitors [105]. The implications of PI3K/AKT signaling in PCa are detailed in Figure 3.

In the context of PCa, a variety of new drugs targeting deregulation of the PI3K/AKT pathway have been developed. Natural products such as Ethanolic Neem Leaf Extract (ENLE) [106], *β*-Caryophyllene Oxide [107], and Dietary flavonoid fisetin [108] have been described as having anti-PI3K/AKT activity in PCa cells. Other drugs, such as curcumin, can inhibit several signaling pathways including AKT [109–112]. Synthetic drugs, such as KN-93, can inhibit PCa cell growth in an androgen-independent manner, by activation and production of reactive oxygen species (ROS), which prevent AKT activation [113]. Other drugs, like GDC-0980, can inhibit PCa cell proliferation through direct inhibition of class I PI3K and mTORC1/2 [114]. HIF-1 proteins are regulators of transcriptional responses against hypoxia and equally important in angiogenesis and tumor growth. An HIF-1*α* inhibitor has been described to inhibit the PI3K/AKT pathway in PCa cell lines [115]. Another example is Gambogic Acid, which limits PCa development through inhibition of both PI3K/AKT and NF-*κ*B pathways [116]. Several mTOR inhibitors have been tested to control the development of androgen-independent PCa [117]. It should be noted that there are currently several AKT inhibitors in clinical trials [118]. For instance, Celecoxib, an inhibitor of cyclooxygenase 2 (COX-2), is described to prevent AKT phosphorylation by inactivating its upstream kinase PDK1 [119]. Perifosine, a phospholipid analogue, can also arrest PCa cell cycle in G1/S or G2/M through AKT inhibition, although the mechanism of inactivation is still not fully understood (but possibly in a PDK1-independent manner) [120]. Genistein, a natural soy-based isoflavone, can inhibit AKT directly, subsequently inhibiting NF-*κ*B activation and inducing apoptosis of PCa cells [121]. On the other hand, the deregulated PI3K/AKT pathway during PCa progression appears to be a reason for the resistance against some anticancer drugs; an example is the resistance to sunitinib in CRPCa, which is correlated with the loss of PTEN expression [122].

## 5. The JAK/STAT Pathway in Prostate Cancer

### 5.1. Pathway Description

Janus Kinase/signal transducers and activators of transcription (JAK/STAT) pathway is recognized as an important membrane-to-nucleus cascade, which may be activated by a wide variety of stimuli such as reactive oxygen species, cytokines, and growth factors [123–127]. JAK/STAT is one of the main cascades required for normal development and cell homeostasis, as well as in the control of cell proliferation, differentiation, cell migration, and apoptosis [124]. Specifically, this pathway is essential to regulate many physiopathological processes including hematopoiesis, gland development, immune response, adipogenesis, and sexually dimorphic growth [128, 129]. Briefly, the signaling activation occurs when specific inducers (e.g., IL-6) binds to and induces the oligomerization of respective receptor subunits (e.g., cytokine receptors), leading to signal propagation by phosphorylation of the receptor-associated tyrosine kinases, known as JAK1-3 and Tyk2 [130, 131]. Particularly, JAK activation occurs when the receptor subunit comes into close proximity (after ligand recognition) and allows the cross-phosphorylation of these tyrosine kinases. Subsequently, activated JAKs induce the phosphorylation of the receptor that now serves as a docking site for additional JAK targets including their major substrates known as signal transducer and activator transcription factors (STATs). STAT proteins have a dual function of signal transduction and transcription activation downstream of phosphorylation events. Indeed, STAT phosphorylation permits the dimerization of other STATs, culminating with the translocation to the nucleus mediated by importin *α*-5 and the Ran nuclear import system. Inside the nucleus, the dimerized STATs bind to specific regulatory sequences along the DNA, leading to activation or repression of target genes [132–135].

### 5.2. Pathway Disruptions Associated with PCa and Therapeutic Targets

The family of STAT transcription factors is constitutively activated in many human tumors. In this sense, these proteins control various cellular events such as proliferation, differentiation, and cell survival. Extensive studies have indicated that this pathway is upregulated in a broad range of cancers [136–140]. A particular member, STAT3, has been shown to be constitutively active in a number of human tumor cell lines as well as primary tumors, including haematological malignancies [141]. For instance, constitutive activation of STAT3 has been related to breast cancer susceptibility cancer 1 (BRCA1) expression in certain tumor cell lines [142]. Additionally, mutations in BRCA genes have been shown to increase predisposition to breast, ovarian, and prostate cancers [139, 143–145]. Both BRCA1 and BRCA2 are related to biological processes including DNA repair, control of cell-cycle checkpoint, and transcriptional regulation. Specifically, BRCA1 performs distinct but more general functions, working as a sensor/signal transducer and as an effector component in response to DNA damage by homologous recombination, while BRCA2 function is more restricted to DNA repair, modulating the activation of RAD51 recombinase, which is also required for homologous recombination [139, 146, 147]. It has been demonstrated that in PCa cells, BRCA1 interacts with JAK1/2, leading to STAT3 phosphorylation and culminating in the induction of cell proliferation and inhibition of apoptotic cell death [142].

STAT3 also targets other genes associated with cell cycle regulation [141, 148]. Upregulation of antiapoptotic STAT3 induces a subset of Bcl-related genes, including Bcl-2, Bcl-XL, Survivin, and Mcl-1, which have been described in PCa and many other tumors [141]. Another STAT3 target gene is the proangiogenic vascular endothelial growth factor (VEGF), involved in tumor invasion and spreading, which directly regulates several matrix metalloproteinases enzymes implicated in tumor cell invasion [141, 149–152]. Moreover, high levels of STAT3 in both malignant and normal tissues adjacent to the tumor have been detected, suggesting that STAT3 activation may occur before any detectable histological changes in the prostate [153]. Additionally, the inhibition of JAK/STAT3 signaling suppresses PCa cell growth and induces apoptosis [154]. In fact, STAT3 inhibition has been suggested as a good strategy to promote the control of cell proliferation and, consequently, tumor growth and metastasis formation [155].

**Figure 4:**
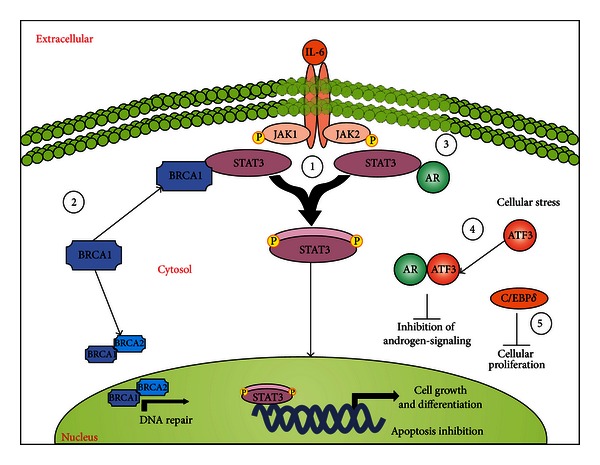
The JAK/STAT signaling in prostate cancer. (1) The JAK/STAT pathway has been found constitutively activated in PCa cells, leading to induction of tumor cell proliferation and apoptosis inhibition mediated by STAT3 activation. (2) BRCA1/2 is required for DNA repair in normal cells. However, in PCa, BRCA1 can bind STAT3 to promote JAK/STAT3 activation. (3) AR is a well-characterized cross-talk pathway in PCa. When activated, AR can bind to STAT3 leading to the activation of JAK/STAT cascade, being important in the induction of cell proliferation and apoptosis inhibition. (4) Under stress conditions, ATF3 is activated and plays a crucial role in the maintenance of cell integrity and homeostasis. ATF3 does so by interacting with AR, leading to inhibition of androgen signaling and, consequently, the inhibition of cell proliferation. However, ATF3 is downregulated in PCa cells, suggesting that this pathway provides an important mechanism of defense against cancer. (5) Similarly, C/EBP*δ* is required to inhibit cell proliferation by binding to STAT3. Nevertheless, C/EBP*δ* is typically downregulated in PCa, and, therefore, it could be used as an strategy in the development of therapeutic drugs against PCa growth.

IL-6 is another factor that has been found to be upregulated in the serum of PCa patients. IL-6 signaling is important to modulate cell growth and differentiation and immune-mediated resistance against infections. Unbalanced IL-6 production has a role in several diseases, such as osteoporosis, atherosclerosis, autoimmune disorders, rheumatoid arthritis, psoriasis, diabetes, and cancer [156, 157]. Several studies have indicated an important role of IL-6 in promoting PCa progression. PCa cells have upregulated expression of both IL-6 and its receptor IL-6R [158], as well as elevated circulating levels of IL-6 in patients with metastatic PCa and CRPCa [159–161], correlating IL-6 production to cancer morbidity [162–164] and differential autocrine and paracrine modulation of PCa cell lines [141, 164–166]. It has been shown that silencing of IL-6 expression by small-interfering RNA in PCa cell lines dramatically decreases cell growth, and this event is accompanied by downregulation of Bcl-2, Bcl-xL, and phosphorylation of AKT, MAPK, and STAT3 both *in vivo* and *in vitro* [167]. Upon IL-6 stimulation, androgen-responsive PCa cell lines also activate STAT3, which further binds to the C/EBP*δ* promoter region, inducing its expression. C/EBP*δ* is a member of the CCAAT/enhancer binding protein (C/EBP) family of transcription factors and plays a crucial role in the regulation of cell growth and fate [168–170]. In fact, C/EBP*δ* overexpression leads to inhibition of tumor growth in PCa [171]. On the other hand, after treatment with IL-6, androgen-independent PCa cells do not exhibit increased C/EBP*δ* gene expression or growth inhibition [171]. However, in PCa patients, the expression of C/EBP*δ* is significantly reduced in metastases when compared to primary PCa [172]. Altogether, the induction of C/EBP*δ* overexpression may function as an alternative of prevention and/or treatment of PCa. The implications of JAK/STAT pathway in PCa are detailed in Figure 4.

## 6. The MAPK Pathway in Prostate Cancer

### 6.1. Pathway Description

Mitogen activated protein kinases (MAPKs) comprise a family of kinases that have a major role in tumor growth and metastasis [173–175]. MAPKs can be divided into three subfamilies: the extracellular-signal-regulated kinases (ERKs), the c-Jun N-terminal kinases (JNKs), and p38 MAPKs that, together with the JNKs, compose the stress-activated protein kinase pathways [175]. All MAPKs have been linked to the regulation of intracellular metabolism, gene expression, cell growth and differentiation, apoptosis, and stress response [176–182]. There is a great body of evidence indicating that alterations in the regulation of MAPKs are extremely important in cancer development [183].

**Figure 5:**
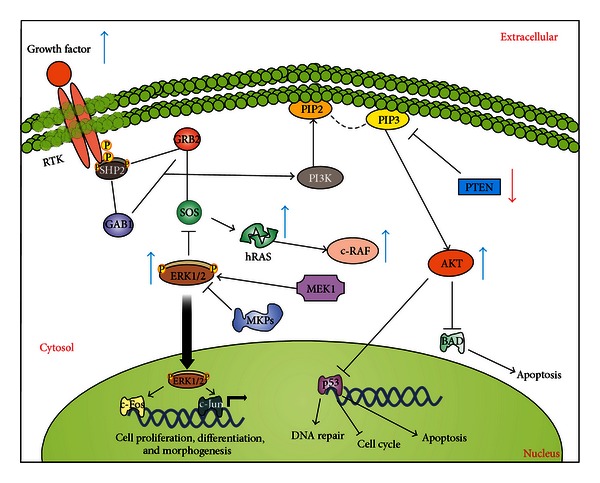
Overview of ERK and PI3K activation and their crosstalk. The binding of the ligand to RTK dimerizes and activates the receptor, leading to the recruitment of multiple Grb2 and Shp2 molecules, which further leads to the binding of a second anchoring protein Gab1 to the complex and to the activation of Son of Sevenless (SOS). This event leads to the activation of Raf/Ras/MEK/ERK pathway. Once phosphorylated, ERKs also phosphorylate a great number of substrates present in both nucleus and cytoplasm. In the nucleus, ERK phosphorylates a series of transcription factors including Elk1, c-Fos, p53, Ets1/2, and c-Jun, each one acting as regulators of cell proliferation, differentiation, and morphogenesis. The recruitment and activation of Grb2 and Shp2 also leads to the recruitment of another docking protein, Gab1. Once phosphorylated, Gab1 recruits PI3K to the membrane, where it phosphorylates the inositol ring of PIP-2 into PIP-3. PIP-3 facilitates the phosphorylation of AKT, which in turn regulates the activity of p53 and BAD. Blue and red arrows indicate up- and downregulated proteins in PCa, respectively.

A plethora of extracellular signals initiate MAPK signaling by the binding and activation of receptor tyrosine kinases (RTKs) or G-protein coupled receptors (GPCRs) (Figure 5). In the case of ERK, the activation through these receptors leads to the recruitment of downstream effectors including growth factor receptor-bound protein 2 (Grb2) and protein tyrosine phosphatase non-receptor type 11 (PTPN11/Shp2), leading to the recruitment of Gab1 (GRB2-associated binding protein 1) and SOS (Son of Sevenless). Then, SOS protein exchanges the GDP in the Ras G-protein for a GTP. The Ras-GTP complex is able to activate the RAF kinase, a MAP-kinase-kinase-kinase (MAP3K) that is an upstream component of the ERK pathway, which in turn phosphorylates the MEK kinase (MAP2K) and, subsequently, phosphorylates and activates the next pathway component MAPK/ERK [184]. The RTKs that interact with Ras, or other members of its superfamily, are diverse and include the epidermal growth factor receptor (EGFR), c-Kit, platelet-derived growth factor receptor (PDGFR), vascular endothelial growth factor receptor (VEGFR), fibroblast growth factor receptor (FGFR), and fms-related tyrosine kinase-3 (FLT-3) [185]. JNKs can be activated by the upstream MKK4 and MKK7 kinases [186, 187]. Although there are many JNK substrates, it is still challenging to identify the molecular networks regulated by the individual JNK family members. It has been found that JNK signaling can alternatively lead to apoptosis or cell survival [187–189]. Downstream targets of the MAPKs include c-Jun, c-Fos, and p53 [184]. c-Jun and c-Fos form a complex called AP-1 (activator protein 1) that acts as a transcription factor. MAPKs are able to translocate to the nucleus and then phosphorylate AP-1 transcription factors (c-Fos, c-Jun, ATF, and JDP family members) to mediate expression of target genes containing a TPA DNA response element (TRE) [190, 191].

### 6.2. Pathway Disruptions Associated with PCa and Therapeutic Targets

MAPK/ERK pathway is shown to be activated in PCa, especially in later stages of the disease, and is often deregulated with AKT signaling [192–194]. The upstream events that lead to activation of MAPK signaling are not well defined but are possibly related to aberrant growth factor signaling [195]. Although members of the Ras family are rarely mutated in PCa [22], Ras and the MEK/ERK pathway are stimulated by EGF, IGF-1, KGF, and FGFs, which are often overexpressed in PCa [196–198]. The expression of Ras or its effector-loop mutants reduces the androgen-dependent requirement of LNCaP cells for growth and increases their PSA expression and tumorigenicity, whereas dominant negative N17-Ras can restore androgen sensitivity to the CRPCa C4-2 cell line [22, 199]. Notably, expression of activated forms of Ras or Raf in the mouse prostate epithelium results in PCa formation [200, 201]. Interestingly, a small percentage of aggressive PCa contains chromosome translocations involving b- or c-Raf, which results in a constitutively activated hybrid protein due to the loss of the N-terminal RAS binding domain [202], which suggests that perturbations of Ras or Raf signaling may occur in PCa through mechanisms other than activating mutations. Also, p38 signals play an important role in the adaptation of malignant cells to hypoxia by increasing the expression of the pore-forming proteins Aquaporins [203] and also by the increased resistance to apoptosis by overexpression of COX-2 [204].

MAPK and its upstream signals seem to be involved not only in PCa but also in the correct development of the prostate. For instance, FGFR2 is an RTK capable of recruiting Grb2 and Shp2 when activated, which acts as an upstream activator of the MAPK signaling pathway [205]. It has been demonstrated that FGFR2 is necessary for the embryological formation of the prostate [205]. Null mutants for *Fgf10* mostly lack prostate budding [206], while conditional deletion of *FGFR2* or *Frs2*α**, a downstream signaling component in prostate epithelium, results in defects in branching morphogenesis [207, 208] It has been also demonstrated that ERK 1/2 is rapidly activated in the urogenital sinus (UGS) when induced by FGF10, and the inhibition of FGFR activity mostly inactivates phosphorylated ERK 1/2 in the UGS, suggesting that FGF10 may signal through MAPK pathway [209]. 

Simultaneous activation of the ERK and AKT signaling pathways has been shown to promote PCa and CRPCa both *in vitro* and *in vivo*, while combined inhibition of these pathways blocks cell proliferation and leads to Bcl-2 and Bim upregulation [192, 210] (Figure 5). Therefore, the MAPK signaling pathway may be a target for PCa therapy, especially if its modulation could be achieved concomitant with other pathways, including PI3K/AKT signaling. The aim of future studies in this area might be directed toward the factors and mechanisms that account for differential function of JNK, p38, and ERK MAPKs as pro- or anti-tumoral factors. In addition, it has been shown that the AKT/mTOR and MAPK pathways participate in the development of PCa. A therapeutic strategy using both rapamycin (mTOR inhibitor) and PD0325901 (MEK1 inhibitor) is shown to inhibit cell growth in a series of PCa cell lines and also to affect tumor growth in mouse models [192]. These results have been further confirmed [211] using inhibitors of both PI3K/AKT/mTOR and RAS/MEK/ERK pathways. These observations may lead to the development of therapeutic approaches to effectively target the pro-tumoral effects of the MAPK pathways.

## 7. The TGF-*β*/SMAD Signaling Pathway in Prostate Cancer

### 7.1. Pathway Description

**Figure 6:**
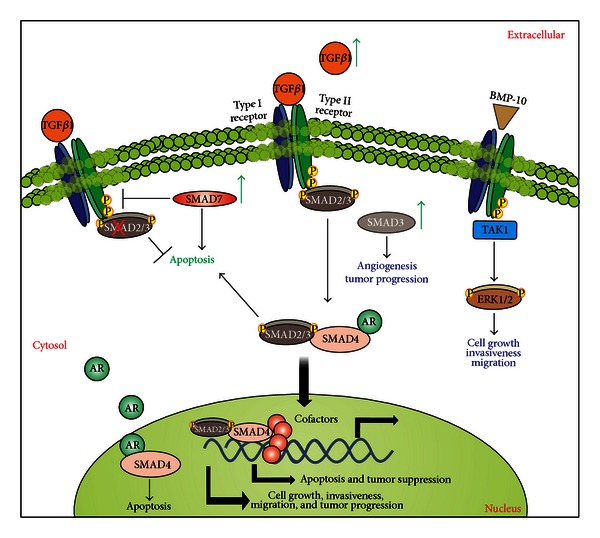
The TGF-*β*/SMAD signaling pathway and its implication in prostate cancer. When a TGF-*β* ligand binds to the constitutively active type II receptor, this complex associates with the type I receptor, forming a tetrameric receptor. The type II receptor phosphorylates and activates the type I receptor, which allows the recruitment of R-SMADs. The activated type I receptor then phosphorylates the MH2 domain of R-SMAD, activating it. Activated R-SMADs form complexes with SMAD4, which is then translocated to the nucleus. In the nucleus, SMAD complexes interact with nuclear proteins to activate or repress the transcription of target genes. Furthermore, BMP-10 can signal through SMAD-independent pathways and inhibit cell growth, invasiveness, and migration. TGF-*β* can also promote androgen receptor (AR) translocation into the nucleus and AR-dependent gene transcription. AR can combine with SMAD4 and regulate TGF-*β*-mediated apoptosis. According to the TGF-*β* central dogma, in normal epithelium or early-stage cancer cells, TGF-*β* acts as a tumor suppressor, by inhibiting cell growth, invasiveness, and motility and promoting apoptosis. In more advanced cancer cells, TGF-*β* has tumor-promoting functions; it promotes proliferation, invasion, and motility of cells and inhibits apoptosis. Green arrows indicate potentially up-regulated proteins in PCa.

The TGF-*β*/SMAD signaling pathway is involved in the regulation of many cellular functions including cell growth, adhesion, migration, cell differentiation, embryonic development, and apoptosis [212]. Accordingly, alterations in the TGF-*β*/SMAD signaling pathway are implicated in many human diseases such as cancer, fibrosis, and several hereditary conditions [213–215]. The pathway initiates when activated ligands (e.g., TGF-*β*) bind to respective receptors, composed of a very diverse cysteine-rich domain, a single-pass transmembrane domain, and a significantly conserved intracellular serine-threonine kinase domain. There are two types of functional receptors that bind to the TGF-*β* ligands, nominated as type I and type II receptors. Type II receptors are constitutively active receptors, and, upon ligand binding, they further activate type I receptors in a phosphorylation-dependent manner. The activated receptors then tetramerize and are able to recruit and activate SMAD proteins, the main effector proteins of this pathway [214–216]. SMADs are intracellular proteins that transduce signals from the TGF-*β* superfamily of ligands to the nucleus, where they activate or suppress the transcription of target genes. There are eight known types of SMADs, which can be divided into three different classes: receptor-regulated SMADs (R-SMADs), common-mediated SMAD (Co-SMAD), and inhibitory SMADs (I-SMADs). Once the receptors are activated, they recruit R-SMADs and phosphorylate them. Phosphorylated R-SMADs can then form complexes with the Co-SMAD SMAD4. This complex is translocated to the nucleus and acts as a transcription factor for many target genes (Figure 6). The I-SMADs, SMAD6 and SMAD7, inhibit SMAD transcriptional activity and the activation of the TGF-*β*/SMAD signaling pathway [214, 216].

### 7.2. Pathway Disruptions Associated with PCa and Therapeutic Targets

Despite the fact that enhanced TGF-*β* levels have been positively associated with prostate cancer progression (Figure 6), TGF-*β*-mediated suppression of growth and motility is also increased in metastatic CRPCa cells, and these events appear to be partially mediated by Smad2/3 signaling [217]. For instance, there is an increased sensitivity to TGF-*β*1-mediated growth inhibition and downregulation of cyclin D in prostate-derived metastatic cell lines C4-2 and C4-2B, when compared to the nonmetastatic cell line (LNCaP cells) and robust phosphorylation and nuclear translocation of Smad2 and Smad3 in metastatic cell lines [217]. The interactions of the stromal environment and epithelial tumor cells apparently dictate PCa progression, and it is likely that TGF-*β* pro-metastatic effects indirectly affects PCa cells through stromal cells, in contrast to its antiproliferative effect on the epithelium [217].

Using a *Cre/flox*-based system in mouse models, it has been observed that, in the absence of TGF-*β*1 produced by activated CD4^+^ T cells and regulatory T cells, there is inhibition of tumor growth and protection from spontaneous PCa [218]. These findings have suggested that TGF-*β*1, produced by activated CD4^+^ T cells, is necessary for tumor evasion from immune surveillance [218]. Furthermore, it is reported that LY2109761, a selective inhibitor of the TGF-*β* type I receptor, provides anti-tumoral effects against PCa cells after growth in bone tissue [219]. In addition, increased volume in normal bone and increased osteoblast and osteoclast numbers are observed after inhibition of the TGF-*β* type I receptor [219]. Thus, TGF-*β*1 has been detected at higher levels in the sera of PCa patients, is associated with bone metastasis, and correlates to a poor clinical outcome [220–222]. Many other studies have also linked changes in the levels of TGF-*β* and of pathway components to cancer progression and to further cellular responses [215, 223, 224].

Evidence for SMAD2 as a critical mediator of TGF-*β*-induced apoptosis has been reported [225]. Silencing of Smad2 expression in NRP-152, a nontumorigenic rat prostate basal epithelial cell line, inhibits TGF-*β*-induced apoptosis. Furthermore, rats injected with small hairpin RNA (shRNA) constructs targeting SMAD2 show palpable PCa tumors in over 80% of the injected sites by day 41 following injection [225].

The activation of the TGF-*β* signaling pathway in an SMAD-independent manner has also been described [226]. BMP-10 (bone morphogenetic protein 10) seems to inhibit growth of PCa cells, mainly by inducing caspase-3 mediated apoptosis and preventing PCa cell migration and invasiveness through SMAD-independent signaling (Figure 6) [226]. BMP-10 overexpression in PCa cells decreases tumor cell growth, cell matrix adhesion, invasion, and migration. These effects seem to be mediated through activation of TAK1 and ERK1/2 [226]. Nodal, another TGF-*β* ligand, has also been found to be overexpressed in some PCa cells and it can be involved in the inhibition of proliferation and induction of migration in these cells [227]. Furthermore, activin A, also known to inhibit growth of PCa cells and promote apoptosis, has been identified as a promoter of bone metastasis in PCa, possibly through SMAD signaling and concomitant elevation of the androgen receptor (AR) gene transcription [228]. Interestingly, the expression of activin A correlates with increased PSA expression, and, therefore, it might be considered as a novel biomarker or potential therapeutic target for the treatment of patients with metastatic PCa [228].

## 8. The Wnt Signaling Pathway in Prostate Cancer

### 8.1. Pathway Description

**Figure 7:**
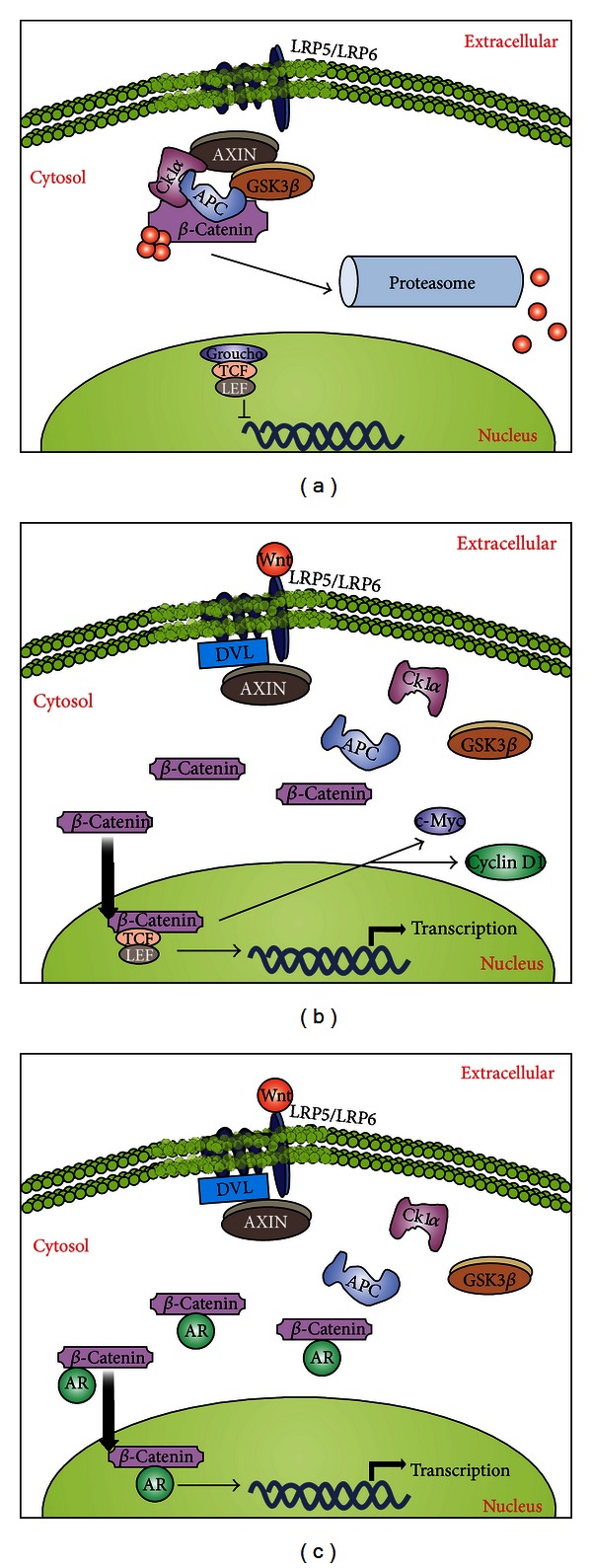
The Wnt signaling and its implications in the development of prostate cancer. (a) In an inactive state, the protein *β*-catenin is sequestered in a complex in the presence of Axin, GSK3*β*, CK1*α*, and APC. This complex allows ubiquitination of *β*-catenin and its subsequent degradation in a proteasome-dependent manner, maintaining this pathway inactive in the absence of Wnt. (b) After binding of Wnt to Frizzled receptor complex (which includes the adaptor molecules LRP5/LRP6), this allows the recruitment of Dishevelled (DVL) and Axin; the recruitment of Axin disrupts the inactivation complex and releases *β*-catenin, which translocates to the nucleus and functions as a transcription factor, inducing expression of several genes related to proliferation, such as *c-myc* and *cyclin D1*. (c) In the PCa environment, *β*-catenin can combine with AR proteins, whose levels are typically increased in PCa, enhancing their function as transcription factors and leading to increased gene expression of pro-survival and proliferative factors.

The Wnt family is composed of a large set of soluble proteins that play important roles in the embryonic developmental processes including cell proliferation, differentiation, and epithelial-mesenchymal interactions [229, 230]. Deregulations in the Wnt pathway have been implicated in cancer development in a variety of tissues including lung, skin, liver, and prostate [229, 231, 232]. Wnt proteins exert their biological effects through two signaling pathways (canonical and non-canonical), which are separated by their ability to stabilize **β*-*catenin [233]. The **β*-*catenin is a multirole protein that promotes cell proliferation by inducing gene transcription through the activation of transcription factors like T cell factor (TCF) and lymphoid enhancer factor (LEF) family of transcription factors [234]. **β*-*Catenin exists in a cytoplasmic complex with Axin, APC (adenomatous polyposis coli gene), and glycogen synthase kinase 3**β** (GSK3**β**), which constitute the “*β*-catenin destruction complex.” In the absence of Wnt, **β*-*catenin is phosphorylated by casein kinase I (CKI*α*) at Ser45; this, in turn, enables GSK3**β** to phosphorylate serine/threonine residues 41, 37, and 33 [235]. Phosphorylation of these last two residues triggers ubiquitination of **β*-*catenin and further degradation by the proteasome ([233, 236, 237], Figure 7(a)). The binding of Wnt proteins to transmembrane Frizzled receptors (FZD) activates the Disheveled protein (DVL), leading to the dephosphorylation of Axin which then reduces the formation of cytoplasmic **β*-*catenin complexes. As a result, free **β*-*catenin accumulates in the cytosol and it is further translocated to the nucleus, where it activates TCF/LEF transcriptional factors ([238], Figure 7(b)). The **β**-catenin/LEF/TCF complexes have been shown to interact with a variety of other nuclear factors to control specific transcriptional targets which include c-Myc, p300, CBP, Hrpt2, Foxo, Bcl9-2, reptin, pontin, c-Jun, Grouchos, Prmt2, CtBP, and cyclin D1 [239–241].

### 8.2. Pathway Disruptions Associated with PCa and Therapeutic Targets

The Wnt family members have been widely studied in PCa progression [241]. It has been hypothesized that PCa cells adopt embryonic signaling pathways that are generally silent in differentiated cells [242]. The role of *β*-catenin in tumorigenesis was first established in colon carcinoma, due to its complex formation with the adenomatous polyposis coli (APC) gene product [243]. APC is a well-known tumor suppressor, which plays a central role in the Wnt signaling pathway by targeting *β*-catenin for degradation. It has been shown that the APC gene is downregulated due to promoter hypermethylation [244], while **β*-*catenin is frequently mutated to an active form [245] and it is typically found in early stages of prostate tumor formation. Indeed, APC exerts a variety of growth regulatory functions that, if disrupted, might lead to tumor formation [235]. A mouse model in which the APC gene has been inactivated results in PCa and adenocarcinoma [246]. Alterations in the APC gene are rare, although loss of heterozygosity and mutation have been detected in some PCa samples [243, 247]. As indicated, some studies have identified the genes *c-Myc* and *cyclin D1* as transcriptional targets activated by the *β-*catenin signaling pathway [248, 249]. The overexpression of *c-Myc* and *cyclin D1* increase cell growth and tumorigenicity in PCa cells, and these genes are apparently activated at the earliest phases of PCa progression [248, 249]. Noticeably, Wnt ligands are up-regulated in PCa, and their expression often correlates with aggressiveness and metastasis [250]. It has been determined that 15 out of the 19 Wnt proteins are expressed in PCa cell lines [251]. Several Wnt ligands, such as Wnt-5a and Wnt-11, can induce the **β*-*catenin-independent (non-canonical) pathway [252]. In particular, Wnt-11 is a secreted protein that modulates cell growth, differentiation, and morphogenesis during development; however, the prevalence of increased expression of Wnt-11 in tumours and the functions of Wnt-11 in PCa cells are not fully understood [253]. Recent data have shown an upregulation of Wnt-11 expression in a significant proportion of PCa tumors, and it has been positively correlated to higher Gleason scores as well as increased PSA levels [254]. Another Wnt ligand, Wnt-4, is highly expressed during embryonic development but is significantly less abundant in the adult prostate [251], suggesting that Wnt signaling might be temporally regulated during prostate development and that it can induce changes in cell fate for prostate progenitors.

Overexpression of Wnt ligands and high levels of **β*-*catenin gene expression have been associated with advanced PCa *in vitro* [255]. Moreover, detection of mutant forms of *β-*catenin has been discovered in PCa [256, 257]. A series of studies have demonstrated that mutant forms of **β*-*catenin that affect GSK3**β**-dependent phosphorylation site (which prevents its degradation and then allows its accumulation in the cytosol) are found in 5%–7% of radical prostatectomy specimens ([250, 258, 259], Figure 7(c)). Another mechanism for increased **β**-catenin expression in PCa may be loss of PTEN, which is common in advanced PCa and results in activation of the PI3K and downstream AKT signaling pathways [260]. AKT can phosphorylate and inactivate GSK3**β**, leading to stabilization and increased levels of **β**-catenin. Indeed, GSK3**β** suppression and subsequent **β**-catenin stabilization have been directly demonstrated in PTEN-deficient PCa cell lines [261]. Consistently, other members of the Wnt pathway are also deregulated in PCa [262]. For instance, Frizzled-4 (FZD4, a Wnt receptor) is co-expressed in human PCa samples with the ETS-related gene (ERG). Gene fusions involving ETS transcription factors (mostly ERG) are found in roughly 50% of all PCas [254]. Further experiments have shown that FZD4 overexpression in ERG-positive PCa leads to an epithelial-to-mesenchymal transition, which is a crucial step in metastasis initiation [254].

In summary, there are several ways that the Wnt pathway can be abnormally activated in cancer, due to the large number of proteins involved in this pathway [257]. For this reason, there is a great potential for the development of a wide array of Wnt antagonists. Several pharmaceutical and biotechnology companies have substantial programs designed to target this pathway [260], and a variety of drugs targeting Wnt pathway are currently on the market or under development [263, 264]. Some categories of drugs include non-steroidal anti-inflammatory drugs (NSAIDs) [265], vitamin D derivatives [266], antibody-based treatments [259], and other small molecule inhibitors [266, 267].

## 9. Conclusions

In the past several decades, an abundance of data related to the signaling events that trigger and maintain PCa have been collected. An increasing knowledge of the interconnections (crosstalks) of different signaling cascades, that ultimately promote the advance of PCa, is of seminal importance for the development of specific drugs which might promote the blockage and/or induction of specific molecules that could lead to the control of tumor progression. In fact, several drugs are currently in clinical trials or being tested in animal models, most of them acting as specific inhibitors of deregulated signaling pathways, such as those described in this review. Nevertheless, a more detailed and interactive panel of the external factors capable of inducing the deregulation(s) observed in the PCa microenvironment is still missing. Thus, it is crucial to pursue a more complete understanding of the cascade-dependent signals that lie behind PCa induction, to consequently lead to the development of fully functional strategies against PCa. This will also advance our knowledge towards more efficient screenings of PCa predisposition, which will certainly lead to increased prevention schemes and early treatments against this malady.

## References

[1] Verras M, Sun Z (2006). Roles and regulation of Wnt signaling and *β*-catenin in prostate cancer. *Cancer Letters*.

[2] Mullins JK, Loeb S (2012). Environmental exposures and prostate cancer. *Urologic Oncology*.

[3] Siegel R, DeSantis C, Virgo K (2012). Cancer treatment and survivorship statistics, 2012. *CA: A Cancer Journal for Clinicians*.

[4] Maluf FC, Smaletz O, Herchenhorn D (2012). Castration-resistant prostate cancer: systemic therapy in 2012. *Clinics*.

[5] Center MM, Jemal A, Lortet-Tieulent J (2012). International variation in prostate cancer incidence and mortality rates. *European Urology*.

[6] Arcangeli G, Saracino B, Gomellini S (2010). A Prospective phase III randomized trial of hypofractionation versus conventional fractionation in patients with high-risk prostate cancer. *International Journal of Radiation Oncology Biology Physics*.

[7] Sim HG, Cheng CWS (2005). Changing demography of prostate cancer in Asia. *European Journal of Cancer*.

[8] Reuben SH (2010). Reducing environmental cancer risk: what we can do now. *The President's Cancer Panel*.

[9] Leitzmann MF, Rohrmann S (2012). Risk factors for the onset of prostatic cancer: age, location, and behavioral correlates. *Clinical Epidemiology*.

[10] Timms BG (2008). Prostate development: a historical perspective. *Differentiation*.

[11] Kristal AR, Arnold KB, Neuhouser ML (2010). Diet, supplement use, and prostate cancer risk: results from the prostate cancer prevention trial. *American Journal of Epidemiology*.

[12] Su LJ, Arab L, Steck SE (2011). Obesity and prostate cancer aggressiveness among African and Caucasian Americans in a population-based study. *Cancer Epidemiology Biomarkers and Prevention*.

[13] Koifman S, Koifman RJ (2003). Environment and cancer in Brazil: an overview from a public health perspective. *Mutation Research*.

[14] Sichieri R, Everhart JE, Mendonca GA (1996). Diet and mortality from common cancers in Brazil: an ecological study. *Cadernos de Saúde Pública*.

[15] Cocco P (2002). On the rumors about the silent spring. Review of the scientific evidence linking occupational and environmental pesticide exposure to endocrine disruption health effects. *Cadernos de Saúde Pública*.

[16] van der Rhee HJ, de Vries E, Coebergh JWW (2006). Does sunlight prevent cancer? A systematic review. *European Journal of Cancer*.

[17] Freedland SJ, Isaacs WB, Platz EA (2005). Prostate size and risk of high-grade, advanced prostate cancer and biochemical progression after radical prostatectomy: a search database study. *Journal of Clinical Oncology*.

[18] Lynch HT, Lynch JF (1996). The Lynch syndrome: melding natural history and molecular genetics to genetic counseling and cancer control. *Cancer Control*.

[19] Jin JK, Dayyani F, Gallick GE (2011). Steps in prostate cancer progression that lead to bone metastasis. *International Journal of Cancer*.

[20] Lonergan PE, Tindall DJ (2011). Androgen receptor signaling in prostate cancer development and progression. *Journal of Carcinogenesis*.

[21] Boorjian SA, Eastham JA, Graefen M (2012). A critical analysis of the long-term impact of radical prostatectomy on cancer control and function outcomes. *European Urology*.

[22] Culig Z, Hobisch A, Cronauer MV (1994). Androgen receptor activation in prostatic tumor cell lines by insulin- like growth factor-I, keratinocyte growth factor, and epidermal growth factor. *Cancer Research*.

[23] Wolk A, Mantzoros CS, Andersson SO (1998). Insulin-like growth factor 1 and prostate cancer risk: a population-based, case-control study. *Journal of the National Cancer Institute*.

[24] Gioeli D, Ficarro SB, Kwiek JJ (2002). Androgen receptor phosphorylation. Regulation and identification of the phosphorylation sites. *Journal of Biological Chemistry*.

[25] Guo Z, Dai B, Jiang T (2006). Regulation of androgen receptor activity by tyrosine phosphorylation. *Cancer Cell*.

[26] Hernes E, Fosså, Berner A, Otnes B, Nesland JM (2004). Expression of the epidermal growth factor receptor family in prostate carcinoma before and during androgen-independence. *British Journal of Cancer*.

[27] Green SM, Mostaghel EA, Nelson PS (2012). Androgen action and metabolism in prostate cancer. *Molecular and Cellular Endocrinology*.

[28] Yu SQ, Lai KP, Xia SJ, Chang HC, Chang C, Yeh S (2009). The diverse and contrasting effects of using human prostate cancer cell lines to study androgen receptor roles in prostate cancer. *Asian Journal of Andrology*.

[29] GIampietri C, Petrungaro S, Padula F (2012). Autophagy modulators sensitize prostate epithelial cancer cell lines to TNF-alpha-dependent apoptosis. *Apoptosis*.

[30] Huang X, Yuan F, Liang M (2012). M-HIFU inhibits tumor growth, suppresses STAT3 activity and enhances tumor specific immunity in a transplant tumor model of prostate cancer. *PLoS ONE*.

[31] Vajda A, Marignol L, Barrett C (2012). Gene expression analysis in prostate cancer: the importance of the endogenous control. *Prostate*.

[32] Lin CE, Chen SU, Lin CC (2012). Lysophosphatidic acid enhances vascular endothelial growth factor-C expression in human prostate cancer PC-3 cells. *PLoS ONE*.

[33] Saraon P, Musrap N, Cretu D (2012). Proteomic profiling of androgen-independent prostate cancer cell lines reveals a role for protein S during the development of high grade and castration-resistant prostate cancer. *Journal of Biological Chemistry*.

[34] Carver BS, Chapinski C, Wongvipat J (2011). Reciprocal feedback regulation of PI3K and androgen receptor signaling in PTEN-deficient prostate cancer. *Cancer Cell*.

[35] Ewald JA, Desotelle JA, Church DR (2012). Androgen deprivation induces senescence characteristics in prostate cancer cells in vitro and in vivo. *Prostate*.

[36] Maxwell PJ, Coulter J, Walker SM (2012). Potentiation of inflammatory CXCL8 signalling sustains cell survival in PTEN-deficient prostate carcinoma. *European Urology*.

[37] Jackson RS, Placzek W, Fernandez A (2012). Sabutoclax, a Mcl-1 antagonist, inhibits tumorigenesis in transgenic mouse and human xenograft models of prostate cancer. *Neoplasia*.

[38] Wang H, Xu Y, Fang Z, Chen S, Balk SP, Yuan X (2012). Doxycycline regulated induction of AKT in murine prostate drives proliferation independently of p27 cyclin dependent kinase inhibitor downregulation. *PLoS ONE*.

[39] Reynolds MA (2008). Molecular alterations in prostate cancer. *Cancer Letters*.

[40] Grossmann ME, Huang H, Tindall DJ (2001). Androgen receptor signaling in androgen-refractory prostate cancer. *Journal of the National Cancer Institute*.

[41] Chan SC, Li Y, Dehm SM (2012). Androgen receptor splice variants activate androgen receptor target genes and support aberrant prostate cancer cell growth independent of canonical androgen receptor nuclear localization signal. *Journal of Biological Chemistry*.

[42] Castoria G, D'Amato L, Ciociola A (2012). Androgen-induced cell migration: role of androgen receptor/filamin A association. *PLoS ONE*.

[43] Waltering KK, Urbanucci A, Visakorpi T (2012). Androgen receptor (AR) aberrations in castration-resistant prostate cancer. *Molecular and Cellular Endocrinology*.

[44] Wang H, Zhang C, Rorick A (2011). CCI-779 inhibits cell-cycle G2-M progression and invasion of castration-resistant prostate cancer via attenuation of UBE2C transcription and mRNA stability. *Cancer Research*.

[45] Urbanucci A, Sahu B, Seppala J (2012). Overexpression of androgen receptor enhances the binding of the receptor to the chromatin in prostate cancer. *Oncogene*.

[46] Shiota M, Yokomizo A, Naito S (2011). Increased androgen receptor transcription: a cause of castration-resistant prostate cancer and a possible therapeutic target. *Journal of Molecular Endocrinology*.

[47] Shiota M, Yokomizo A, Naito S (2011). Oxidative stress and androgen receptor signaling in the development and progression of castration-resistant prostate cancer. *Free Radical Biology and Medicine*.

[48] Zuo Y, Cheng JK (2009). Small ubiquitin-like modifier protein-specific protease 1 and prostate cancer. *Asian Journal of Andrology*.

[49] Bawa-Khalfe T, Cheng J, Wang Z, Yeh ETH (2007). Induction of the SUMO-specific protease 1 transcription by the androgen receptor in prostate cancer cells. *Journal of Biological Chemistry*.

[50] Perkins ND (2007). Integrating cell-signalling pathways with NF-kappaB and IKK function. *Nature Reviews Molecular Cell Biology*.

[51] Cai C, Jiang FN, Liang YX (2011). Classical and alternative nuclear factor-kappaB pathways: a comparison among normal prostate, benign prostate hyperplasia and prostate cancer. *Pathology and Oncology Research*.

[52] Tergaonkar V (2006). NF*κ*B pathway: a good signaling paradigm and therapeutic target. *International Journal of Biochemistry and Cell Biology*.

[53] Razani B, Reichardt AD, Cheng G (2011). Non-canonical NF-kappaB signaling activation and regulation: principles and perspectives. *Immunological Reviews*.

[54] Rickert RC, Jellusova J, Miletic AV (2011). Signaling by the tumor necrosis factor receptor superfamily in B-cell biology and disease. *Immunological Reviews*.

[55] Gu L, Dagvadorj A, Lutz J (2010). Transcription factor Stat3 stimulates metastatic behavior of human prostate cancer cells in vivo, whereas Stat5b has a preferential role in the promotion of prostate cancer cell viability and tumor growth. *American Journal of Pathology*.

[56] Chen J, Giridhar KV, Zhang L, Xu S, Jane Wang Q (2011). A protein kinase C/protein kinase D pathway protects LNCaP prostate cancer cells from phorbol ester-induced apoptosis by promoting ERK1/2 and NF-*κ*B activities. *Carcinogenesis*.

[57] Hsu A, Bruno RS, Löhr CV (2011). Dietary soy and tea mitigate chronic inflammation and prostate cancer via NF*κ*B pathway in the Noble rat model. *Journal of Nutritional Biochemistry*.

[58] Benelli R, Vene R, Ciarlo M, Carlone S, Barbieri O, Ferrari N (2012). The AKT/NF-kappaB inhibitor xanthohumol is a potent anti-lymphocytic leukemia drug overcoming chemoresistance and cell infiltration. *Biochemical Pharmacology*.

[59] Jain G, Voogdt C, Tobias A (2012). IkappaB kinases modulate the activity of the androgen receptor in prostate carcinoma cell lines. *Neoplasia*.

[60] Fang Y, Sun H, Zhai J (2012). Antitumor activity of NF-kB decoy oligodeoxynucleotides in a prostate cancer cell line. *Asian Pacific Journal of Cancer Prevention*.

[61] Xiao W, Hodge DR, Wang L, Yang X, Zhang X, Farrar WL (2004). Co-operative functions between nuclear factors NF*κ*B and CCAT/enhancer-binding protein-*β* (C/EBP-*β*) regulate the IL-6 promoter in autocrine human prostate cancer cells. *Prostate*.

[62] Shukla S, MacLennan GT, Fu P (2004). Nuclear factor-*κ*B/p65 (Rel A) is constitutively activated in human prostate adenocarcinoma and correlates with disease progression. *Neoplasia*.

[63] Chen CD, Sawyers CL (2002). NF-*κ*B activates prostate-specific antigen expression and is upregulated in androgen-independent prostate cancer. *Molecular and Cellular Biology*.

[64] Paz De Miguel M, Royuela M, Bethencourt FR, Santamaría L, Fraile B, Paniagua R (2000). Immunoexpression of tumour necrosis factor-*α* and its receptors 1 and 2 correlates with proliferation/apoptosis equilibrium in normal, hyperplasic and carcinomatous human prostrate. *Cytokine*.

[65] Rodriguez-Berriguete G, Fraile B, Paniagua R, Aller P, Royuela M (2012). Expression of NF-kappaB-related proteins and their modulation during TNF-alpha-provoked apoptosis in prostate cancer cells. *Prostate*.

[66] Bouraoui Y, Ricote M, García-Tuñón I (2008). Pro-inflammatory cytokines and prostate-specific antigen in hyperplasia and human prostate cancer. *Cancer Detection and Prevention*.

[67] Srinivasan S, Kumar R, Koduru S, Chandramouli A, Damodaran C (2010). Inhibiting TNF-mediated signaling: a novel therapeutic paradigm for androgen independent prostate cancer. *Apoptosis*.

[68] Rodríguez-Berriguete G, Fraile B, de Bethencourt FR (2010). Role of IAPs in prostate cancer progression: immunohistochemical study in normal and pathological (benign hyperplastic, prostatic intraepithelial neoplasia and cancer) human prostate. *BMC Cancer*.

[69] Chen CD, Welsbie DS, Tran C (2004). Molecular determinants of resistance to antiandrogen therapy. *Nature Medicine*.

[70] Zhang L, Altuwaijri S, Deng F (2009). NF-*κ*B regulates androgen receptor expression and prostate cancer growth. *American Journal of Pathology*.

[71] Jain G, Cronauer MV, Schrader M, Moller P, Marienfeld RB (2012). NF-kappaB signaling in prostate cancer: a promising therapeutic target?. *World Journal of Urology*.

[72] Grivennikov SI, Karin M (2010). Dangerous liaisons: STAT3 and NF-*κ*B collaboration and crosstalk in cancer. *Cytokine and Growth Factor Reviews*.

[73] Schneider G, Kramer OH (2011). NFkappaB/p53 crosstalk-a promising new therapeutic target. *Biochimica et Biophysica Acta*.

[74] Oeckinghaus A, Hayden MS, Ghosh S (2011). Crosstalk in NF-kappaB signaling pathways. *Nature Immunology*.

[75] Vivanco I, Sawyers CL (2002). The phosphatidylinositol 3-kinase-AKT pathway in human cancer. *Nature Reviews Cancer*.

[76] Bader AG, Kang S, Zhao L, Vogt PK (2005). Oncogenic PI3K deregulates transcription and translation. *Nature Reviews Cancer*.

[77] Engelman JA, Luo J, Cantley LC (2006). The evolution of phosphatidylinositol 3-kinases as regulators of growth and metabolism. *Nature Reviews Genetics*.

[78] Guertin DA, Sabatini DM (2007). Defining the role of mTOR in cancer. *Cancer Cell*.

[79] Bozulic L, Hemmings BA (2009). PIKKing on PKB: regulation of PKB activity by phosphorylation. *Current Opinion in Cell Biology*.

[80] Scheid MP, Woodgett JR (2001). PKB/AKT: functional insights from genetic models. *Nature Reviews Molecular Cell Biology*.

[81] Manning BD, Cantley LC (2007). AKT/PKB signaling: navigating downstream. *Cell*.

[82] Cantley LC (2002). The phosphoinositide 3-kinase pathway. *Science*.

[83] Morgan TM, Koreckij TD, Corey E (2009). Targeted therapy for advanced prostate cancer: inhibition of the PI3K/Akt/mTOR pathway. *Current Cancer Drug Targets*.

[84] Di Cristofano A, Pesce B, Cordon-Cardo C, Pandolfi PP (1998). Pten is essential for embryonic development and tumour suppression. *Nature Genetics*.

[85] Boormans JL, Korsten H, Ziel-Van Der Made ACJ, Van Leenders GJLH, Verhagen PCMS, Trapman J (2010). E17K substitution in AKT1 in prostate cancer. *British Journal of Cancer*.

[86] Trotman LC, Niki M, Dotan ZA (2003). Pten dose dictates cancer progression in the prostate. *PLoS Biology*.

[87] Chen Z, Trotman LC, Shaffer D (2005). Crucial role of p53-dependent cellular senescence in suppression of Pten-deficient tumorigenesis. *Nature*.

[88] Di Cristofano A, De Acetis M, Koff A, Cordon-Cardo C, P Pandolfi P (2001). Pten and p27KIP1 cooperate in prostate cancer tumor suppression in the mouse. *Nature Genetics*.

[89] Poliseno L, Salmena L, Riccardi L (2010). Identification of the miR-106b*∼*25 microRNA cluster as a proto-oncogenic PTEN-targeting intron that cooperates with its host gene MCM7 in transformation. *Science Signaling*.

[90] Song MS, Salmena L, Carracedo A (2008). The deubiquitinylation and localization of PTEN are regulated by a HAUSP-PML network. *Nature*.

[91] Song MS, Salmena L, Pandolfi PP (2012). The functions and regulation of the PTEN tumour suppressor. *Nature Reviews Molecular Cell Biology*.

[92] Almasan A, Ashkenazi A (2003). Apo2L/TRAIL: apoptosis signaling, biology, and potential for cancer therapy. *Cytokine and Growth Factor Reviews*.

[93] Xu J, Zhou JY, Wei WZ, Wu GS (2010). Activation of the Akt survival pathway contributes to TRAIL resistance in cancer cells. *PLoS ONE*.

[94] Tzenaki N, Andreou M, Stratigi K (2012). High levels of p110delta PI3K expression in solid tumor cells suppress PTEN activity, generating cellular sensitivity to p110delta inhibitors through PTEN activation. *FASEB Journal*.

[95] Lee SH, Poulogiannis G, Pyne S (2010). A constitutively activated form of the p110*β* isoform of PI3-kinase induces prostatic intraepithelial neoplasia in mice. *Proceedings of the National Academy of Sciences of the United States of America*.

[96] Cinar B, Fang PK, Lutchman M (2007). The pro-apoptotic kinase Mst1 and its caspase cleavage products are direct inhibitors of Akt1. *EMBO Journal*.

[97] Cinar B, Collak FK, Lopez D (2011). MST1 is a multifunctional caspase-independent inhibitor of androgenic signaling. *Cancer Research*.

[98] Yuan Z, Kim D, Shu S (2010). Phosphoinositide 3-kinase/Akt inhibits MST1-mediated pro-apoptotic signaling through phosphorylation of threonine 120. *Journal of Biological Chemistry*.

[99] Mahajan K, Coppola D, Challa S (2010). Ack1 mediated AKT/PKB tyrosine 176 phosphorylation regulates its activation. *PloS ONE*.

[100] Nacerddine K, Beaudry JB, Ginjala V (2012). Akt-mediated phosphorylation of Bmi1 modulates its oncogenic potential, E3 ligase activity, and DNA damage repair activity in mouse prostate cancer. *Journal of Clinical Investigation*.

[101] Gao M, Patel R, Ahmad I (2012). SPRY2 loss enhances ErbB trafficking and PI3K/AKT signalling to drive human and mouse prostate carcinogenesis. *EMBO Molecular Medicine*.

[102] Adhami VM, Siddiqui IA, Sarfaraz S (2009). Effective prostate cancer chemopreventive intervention with green tea polyphenols in the TRAMP model depends on Thestage of the disease. *Clinical Cancer Research*.

[103] Adhami VM, Siddiqui IA, Ahmad N, Gupta S, Mukhtar H (2004). Oral consumption of green tea polyphenols inhibits insulin-like growth factor-I-induced signaling in an autochthonous mouse model of prostate cancer. *Cancer Research*.

[104] Kung HJ, Evans CP (2009). Oncogenic activation of androgen receptor. *Urologic Oncology*.

[105] Clegg NJ, Couto SS, Wongvipat J (2011). MYC cooperates with AKT in prostate tumorigenesis and alters sensitivity to mTOR inhibitors. *PLoS ONE*.

[106] Gunadharini DN, Elumalai P, Arunkumar R, Senthilkumar K, Arunakaran J (2011). Induction of apoptosis and inhibition of PI3K/Akt pathway in PC-3 and LNCaP prostate cancer cells by ethanolic neem leaf extract. *Journal of Ethnopharmacology*.

[107] Park KR, Nam D, Yun HM (2011). beta-Caryophyllene oxide inhibits growth and induces apoptosis through the suppression of PI3K/AKT/mTOR/S6K1 pathways and ROS-mediated MAPKs activation. *Cancer Letters*.

[108] Adhami VM, Syed DN, Khan N, Mukhtar H (2012). Dietary flavonoid fisetin: a novel dual inhibitor of PI3K/Akt and mTOR for prostate cancer management. *Biochemical Pharmacology*.

[109] Wang Z, Zhang Y, Banerjee S, Li Y, Sarkar FH (2006). Notch-1 down-regulation by curcumin is associated with the inhibition of cell growth and the induction of apoptosis in pancreatic cancer cells. *Cancer*.

[110] Ryu MJ, Cho M, Song JY (2008). Natural derivatives of curcumin attenuate the Wnt/*β*-catenin pathway through down-regulation of the transcriptional coactivator p300. *Biochemical and Biophysical Research Communications*.

[111] Yu S, Shen G, Tin OK, Kim JH, Kong ANT (2008). Curcumin inhibits Akt/mammalian target of rapamycin signaling through protein phosphatase-dependent mechanism. *Molecular Cancer Therapeutics*.

[112] Ślusarz A, Shenouda NS, Sakla MS (2010). Common botanical compounds inhibit the hedgehog signaling pathway in prostate cancer. *Cancer Research*.

[113] Rokhlin OW, Guseva NV, Taghiyev AF, Glover RA, Cohen MB (2010). KN-93 inhibits androgen receptor activity and induces cell death irrespective of p53 and Akt status in prostate cancer. *Cancer Biology and Therapy*.

[114] Wallin JJ, Edgar KA, Guan J (2011). GDC-0980 is a novel class I PI3K/mTOR kinase inhibitor with robust activity in cancer models driven by the PI3K pathway. *Molecular Cancer Therapeutics*.

[115] Manohar SM, Padgaonkar AA, Badhwar AJ (2011). A novel inhibitor of hypoxia-inducible factor-1alpha P3155 also modulates PI3K pathway and inhibits growth of prostate cancer cells. *BMC Cancer*.

[116] Lu L, Tang D, Wang L (2012). Gambogic acid inhibits TNF-alpha-induced invasion of human prostate cancer PC3 cells in vitro through PI3K/Akt and NF-kappaB signaling pathways. *Acta Pharmacologica Sinica*.

[117] Burgio SL, Fabbri F, Seymour IJ, Zoli W, Amadori D, De Giorgi U (2012). Perspectives on mTOR inhibitors for castration-refractory prostate cancer. *Current Cancer Drug Targets*.

[118] Nelson EC, Evans CP, Mack PC, Devere-White RW, Lara PN (2007). Inhibition of Akt pathways in the treatment of prostate cancer. *Prostate Cancer and Prostatic Diseases*.

[119] Granville CA, Memmott RM, Gills JJ, Dennis PA (2006). Handicapping the race to develop inhibitors of the phosphoinositide 3-kinase/Akt/mammalian target of rapamycin pathway. *Clinical Cancer Research*.

[120] Kondapaka SB, Singh SS, Dasmahapatra GP, Sausville EA, Roy KK (2003). Perifosine, a novel alkylphospholipid, inhibits protein kinase B activation. *Molecular Cancer Therapeutics*.

[121] Tepper CG, Vinall RL, Wee CB (2007). GCP-mediated growth inhibition and apoptosis of prostate cancer cells via androgen receptor-dependent and -independent mechanisms. *Prostate*.

[122] Makhov PB, Golovine K, Kutikov A (2012). Modulation of Akt/mTOR signaling overcomes sunitinib resistance in renal and prostate cancer cells. *Molecular Cancer Therapeutics*.

[123] Kiu H, Nicholson SE (2012). Biology and significance of the JAK/STAT signalling pathways. *Growth Factors*.

[124] Harrison DA (2012). The Jak/STAT pathway. *Cold Spring Harbor Perspectives in Biology*.

[125] Li WX (2008). Canonical and non-canonical JAK-STAT signaling. *Trends in Cell Biology*.

[126] Hebenstreit D, Horejs-Hoeck J, Duschl A (2005). JAK/STAT-dependent gene regulation by cytokines. *Drug News and Perspectives*.

[127] O’Shea JJ, Park H, Pesu M, Borie D, Changelian P (2005). New strategies for immunosuppression: interfering with cytokines by targeting the Jak/Stat pathway. *Current Opinion in Rheumatology*.

[128] Igaz P, Tóth S, Falus A (2001). Biological and clinical significance of the JAK-STAT pathway; lessons from knockout mice. *Inflammation Research*.

[129] O'Shea JJ, Gadina M, Schreiber RD (2002). Cytokine signaling in 2002: new surprises in the Jak/Stat pathway. *Cell*.

[130] Darnell JE (1997). STATs and gene regulation. *Science*.

[131] Ihle JN, Thierfelder W, Teglund S (1998). Signaling by the cytokine receptor superfamily. *Annals of the New York Academy of Sciences*.

[132] Shuai K, Horvath CM, Huang LHT, Qureshi SA, Cowburn D, Darnell JE (1994). Interferon activation of the transcription factor Stat91 involves dimerization through SH2-phosphotyrosyl peptide interactions. *Cell*.

[133] Gupta S, Barrett T, Whitmarsh AJ (1996). Selective interaction of JNK protein kinase isoforms with transcription factors. *EMBO Journal*.

[134] Mowen K, David M (1998). Role of the STAT1-SH2 domain and STAT2 in the activation and nuclear translocation of STAT1. *Journal of Biological Chemistry*.

[135] Rawlings JS, Rosler KM, Harrison DA (2004). The JAK/STAT signaling pathway. *Journal of Cell Science*.

[136] Bromberg J (2002). Stat proteins and oncogenesis. *Journal of Clinical Investigation*.

[137] Yu H, Jove R (2004). The stats of cancer—new molecular targets come of age. *Nature Reviews Cancer*.

[138] Tutt A, Ashworth A (2002). The relationship between the roles of BRCA genes in DNA repair and cancer predisposition. *Trends in Molecular Medicine*.

[139] Wallerand H, Robert G, Bernhard JC, Ravaud A, Patard JJ (2010). Tyrosine-kinase inhibitors in the treatment of muscle invasive bladder cancer and hormone refractory prostate cancer. *Archivos Espanoles de Urologia*.

[140] Kwon EM, Holt SK, Fu R (2012). Androgen metabolism and JAK/STAT pathway genes and prostate cancer risk. *Cancer Epidemiology*.

[141] Frank DA (2007). STAT3 as a central mediator of neoplastic cellular transformation. *Cancer Letters*.

[142] Gao B, Shen X, Kunos G (2001). Constitutive activation of JAK-STAT3 signaling by BRCA1 in human prostate cancer cells. *FEBS Letters*.

[143] Venkitaraman AR (2002). Cancer susceptibility and the functions of BRCA1 and BRCA2. *Cell*.

[144] Ostrander EA, Udler MS (2008). The role of the BRCA2 gene in susceptibility to prostate cancer revisited. *Cancer Epidemiology Biomarkers and Prevention*.

[145] Panchal S, Shachar O, O’Malley F (2009). Breast cancer in a BRCA2 mutation carrier with a history of prostate cancer. *Nature Reviews Clinical Oncology*.

[146] Rahman N, Stratton MR (1998). The genetics of breast cancer susceptibility. *Annual Review of Genetics*.

[147] Kerr P, Ashworth A (2001). New complexities for BRCA1 and BRCA2. *Current Biology*.

[148] Sakaguchi M, Oka M, Iwasaki T, Fukami Y, Nishigori C (2012). Role and regulation of STAT3 phosphorylation at Ser727 in melanocytes and melanoma cells. *Journal of Investigative Dermatology*.

[149] Cao J, Kozarekar P, Pavlaki M, Chiarelli C, Bahou WF, Zucker S (2004). Distinct roles for the catalytic and hemopexin domains of membrane type 1-matrix metalloproteinase in substrate degradation and cell migration. *Journal of Biological Chemistry*.

[150] Romanov VI, Whyard T, Adler HL, Waltzer WC, Zucker S (2004). Prostate cancer cell adhesion to bone marrow endothelium: the role of prostate-specific antigen. *Cancer Research*.

[151] Nguyen HL, Zucker S, Zarrabi K, Kadam P, Schmidt C, Cao J (2011). Oxidative stress and prostate cancer progression are elicited by membrane-type 1 matrix metalloproteinase. *Molecular Cancer Research*.

[152] Kesanakurti D, Chetty C, Dinh DH, Gujrati M, Rao JS (2012). Role of MMP-2 in the regulation of IL-6/Stat3 survival signaling via interaction with alpha5beta1 integrin in glioma. *Oncogene*.

[153] Dhir R, Ni Z, Lou W, DeMiguel F, Grandis JR, Gao AC (2002). Stat3 activation in prostatic carcinomas. *Prostate*.

[154] Liu X, He Z, Li CH, Huang G, Ding C, Liu H (2012). Correlation analysis of JAK-STAT pathway components on prognosis of patients with prostate cancer. *Pathology and Oncology Research*.

[155] Aalinkeel R, Hu Z, Nair BB (2010). Genomic analysis highlights the role of the JAK-STAT signaling in the anti-proliferative effects of dietary—flavonoid “ashwagandha” in prostate cancer cells. *Evidence-Based Complementary and Alternative Medicine*.

[156] Ishihara K, Hirano T (2002). IL-6 in autoimmune disease and chronic inflammatory proliferative disease. *Cytokine and Growth Factor Reviews*.

[157] Kishimoto T (2005). Interleukin-6: from basic science to medicine—40 years in immunology. *Annual Review of Immunology*.

[158] Hobisch A, Rogatsch H, Hittmair A (2000). Immunohistochemical localization of interleukin-6 and its receptor in benign, premalignant and malignant prostate tissue. *Journal of Pathology*.

[159] Smith PC, Hobisch A, Lin DL, Culig Z, Keller ET (2001). Interleukin-6 and prostate cancer progression. *Cytokine and Growth Factor Reviews*.

[160] Stark JR, Li H, Kraft P (2009). Circulating prediagnostic interleukin-6 and C-reactive protein and prostate cancer incidence and mortality. *International Journal of Cancer*.

[161] Karkera J, Steiner H, Li W (2011). The anti-interleukin-6 antibody siltuximab down-regulates genes implicated in tumorigenesis in prostate cancer patients from a phase I study. *Prostate*.

[162] Twillie DA, Eisenberger MA, Carducci MA, Hseih WS, Kim WY, Simons JW (1995). Interleukin-6: a candidate mediator of human prostate cancer morbidity. *Urology*.

[163] Okamoto M, Lee C, Oyasu R (1997). Interleukin-6 as a paracrine and autocrine growth factor in human prostatic carcinoma cells in vitro. *Cancer Research*.

[164] Cavarretta IT, Neuwirt H, Untergasser G (2007). The antiapoptotic effect of IL-6 autocrine loop in a cellular model of advanced prostate cancer is mediated by Mcl-1. *Oncogene*.

[165] Chung TD, Yu JJ, Spiotto MT, Bartkowski M, Simons JW (1999). Characterization of the role of IL-6 in the progression of prostate cancer. *Prostate*.

[166] Lou W, Ni Z, Dyer K, Tweardy DJ, Gao AC (2000). Interleukin-6 induces prostate cancer cell growth accompanied by activation of stat3 signaling pathway. *Prostate*.

[167] Sakai I, Miyake H, Terakawa T, Fujisawa M (2011). Inhibition of tumor growth and sensitization to chemotherapy by RNA interference targeting interleukin-6 in the androgen-independent human prostate cancer PC3 model. *Cancer Science*.

[168] Dearth LR, DeWille J (2003). An AU-rich element in the 3′ untranslated region of the C/EBP*δ* mRNA is important for protein binding during G0 growth arrest. *Biochemical and Biophysical Research Communications*.

[169] Dearth LR, DeWille J (2003). Posttranscriptional and posttranslational regulation of C/EBP*δ* in Go growth-arrested mammary epithelial cells. *Journal of Biological Chemistry*.

[170] Ramji DP, Foka P (2002). CCAAT/enhancer-binding proteins: structure, function and regulation. *Biochemical Journal*.

[171] Sanford DC, DeWille JW (2005). C/EBP*δ* is a downstream mediator of IL-6 induced growth inhibition of prostate cancer cells. *Prostate*.

[172] LaTulippe E, Satagopan J, Smith A (2002). Comprehensive gene expression analysis of prostate cancer reveals distinct transcriptional programs associated with metastatic disease. *Cancer Research*.

[173] Binétruy B, Heasley L, Bost F, Caron L, Aouadi M (2007). Concise review: regulation of embryonic stem cell lineage commitment by mitogen-activated protein kinases. *Stem Cells*.

[174] Dhillon AS, Hagan S, Rath O, Kolch W (2007). MAP kinase signalling pathways in cancer. *Oncogene*.

[175] Wagner EF, Nebreda AR (2009). Signal integration by JNK and p38 MAPK pathways in cancer development. *Nature Reviews Cancer*.

[176] Ip YT, Davis RJ (1998). Signal transduction by the c-Jun N-terminal kinase (JNK)—from inflammation to development. *Current Opinion in Cell Biology*.

[177] Holmström TH, Schmitz I, Söderström TS (2000). MAPK/ERK signaling in activated T cells inhibits CD95/Fas-mediated apoptosis downstream of DISC assembly. *EMBO Journal*.

[178] Gupta K, Kshirsagar S, Li W (1999). VEGF prevents apoptosis of human microvascular endothelial cells via opposing effects on MAPK/ERK and SAPK/JNK signaling. *Experimental Cell Research*.

[179] Ogata A, Chauhan D, Teoh G (1997). IL-6 triggers cell growth via the ras-dependent mitogen-activated protein kinase cascade. *Journal of Immunology*.

[180] Flati V, Pasini E, D’Antona G, Speca S, Toniato E, Martinotti S (2008). Intracellular mechanisms of metabolism regulation: the role of signaling via the mammalian target of rapamycin pathway and other routes. *American Journal of Cardiology*.

[181] Davis S, Vanhoutte P, Pagès C, Caboche J, Laroche S (2000). The MAPK/ERK cascade targets both Elk-1 and cAMP response element- binding protein to control long-term potentiation-dependent gene expression in the dentate gyrus in vivo. *Journal of Neuroscience*.

[182] Guicheux J, Lemonnier J, Ghayor C, Suzuki A, Palmer G, Caverzasio J (2003). Activation of p38 mitogen-activated protein kinase and c-Jun-NH2-terminal kinase by BMP-2 and their implication in the stimulation of osteoblastic cell differentiation. *Journal of Bone and Mineral Research*.

[183] Huang C, Jacobson K, Schaller MD (2004). MAP kinases and cell migration. *Journal of Cell Science*.

[184] Lemmon MA, Schlessinger J (2010). Cell signaling by receptor tyrosine kinases. *Cell*.

[185] Fecher LA, Amaravadi RK, Flaherty KT (2008). The MAPK pathway in melanoma. *Current Opinion in Oncology*.

[186] Fleming Y, Armstrong CG, Morrice N, Paterson A, Goedert M, Cohen P (2000). Synergistic activation of stress-activated protein kinase 1/c-Jun N-terminal kinase (SAPK1/JNK) isoforms by mitogen-activated protein kinase kinase 4 (MKK4) and MKK7. *Biochemical Journal*.

[187] Haeusgen W, Herdegen T, Waetzig V (2011). The bottleneck of JNK signaling: molecular and functional characteristics of MKK4 and MKK7. *European Journal of Cell Biology*.

[188] Xin W, Yun KJ, Ricci F (2004). MAP2K4/MKK4 expression in pancreatic cancer: genetic validation of immunohistochemistry and relationship to disease course. *Clinical Cancer Research*.

[189] Yamada SD, Hickson JA, Hrobowski Y (2002). Mitogen-activated protein kinase kinase 4 (MKK4) acts as a metastasis suppressor gene in human ovarian carcinoma. *Cancer Research*.

[190] Chiu R, Boyle WJ, Meek J, Smeal T, Hunter T, Karin M (1988). The c-Fos protein interacts with c-Jun/AP-1 to stimulate transcription of AP-1 responsive genes. *Cell*.

[191] Jonat C, Rahmsdorf HJ, Park KK (1990). Antitumor promotion and antiinflammation: down-modulation of AP-1 (Fos/Jun) activity by glucocorticoid hormone. *Cell*.

[192] Kinkade CW, Castillo-Martin M, Puzio-Kuter A (2008). Targeting AKT/mTOR and ERK MAPK signaling inhibits hormone-refractory prostate cancer in a preclinical mouse model. *Journal of Clinical Investigation*.

[193] Abreu-Martin MT, Chari A, Palladino AA, Craft NA, Sawyers CL (1999). Mitogen-activated protein kinase kinase kinase 1 activates androgen receptor-dependent transcription and apoptosis in prostate cancer. *Molecular and Cellular Biology*.

[194] Gioeli D, Mandell JW, Petroni GR, Frierson HF, Weber MJ (1999). Activation of mitogen-activated protein kinase associated with prostate cancer progression. *Cancer Research*.

[195] Carey AM, Pramanik R, Nicholson LJ (2007). Ras-MEK-ERK signaling cascade regulates androgen receptor element-inducible gene transcription and DNA synthesis in prostate cancer cells. *International Journal of Cancer*.

[196] Dorkin TJ, Robinson MC, Marsh C, Bjartell A, Neal DE, Leung HY (1999). FGF8 over-expression in prostate cancer is associated with decreased patient survival and persists in androgen independent disease. *Oncogene*.

[197] Steiner H, Godoy-Tundidor S, Rogatsch H (2003). Accelerated in vivo growth of prostate tumors that up-regulate interleukin-6 is associated with reduced retinoblastoma protein expression and activation of the mitogen-activated protein kinase pathway. *American Journal of Pathology*.

[198] Bakin RE, Gioeli D, Bissonette EA, Weber MJ (2003). Attenuation of Ras signaling restores androgen sensitivity to hormone-refractory C4-2 prostate cancer cells. *Cancer Research*.

[199] Jeong JH, Wang Z, Guimaraes AS (2008). BRAF activation initiates but does not maintain invasive prostate adenocarcinoma. *PLoS ONE*.

[200] Pearson HB, Phesse TJ, Clarke AR (2009). K-ras and Wnt signaling synergize to accelerate prostate tumorigenesis in the mouse. *Cancer Research*.

[201] Palanisamy N, Ateeq B, Kalyana-Sundaram S (2010). Rearrangements of the RAF kinase pathway in prostate cancer, gastric cancer and melanoma. *Nature Medicine*.

[202] Tie L, Lu N, Pan XY (2012). Hypoxia-induced up-regulation of aquaporin-1 protein in prostate cancer cells in a p38-dependent manner. *Cellular Physiology and Biochemistry*.

[203] Limami Y, Pinon A, Leger DY (2012). The P2Y2/Src/p38/COX-2 pathway is involved in the resistance to ursolic acid-induced apoptosis in colorectal and prostate cancer cells. *Biochimie*.

[204] Katoh M (2009). FGFR2 abnormalities underlie a spectrum of bone, skin, and cancer pathologies. *Journal of Investigative Dermatology*.

[205] Marker PC, Donjacour AA, Dahiya R, Cunha GR (2003). Hormonal, cellular, and molecular control of prostatic development. *Developmental Biology*.

[206] Donjacour AA, Thomson AA, Cunha GR (2003). FGF-10 plays an essential role in the growth of the fetal prostate. *Developmental Biology*.

[207] Lin Y, Liu G, Zhang Y (2007). Fibroblast growth factor receptor 2 tyrosine kinase is required for prostatic morphogenesis and the acquisition of strict androgen dependency for adult tissue homeostasis. *Development*.

[208] Zhang Y, Zhang J, Lin Y (2008). Role of epithelial cell fibroblast growth factor receptor substrate 2*α* in prostate development, regeneration and tumorigenesis. *Development*.

[209] Kuslak SL, Marker PC (2007). Fibroblast growth factor receptor signaling through MEK-ERK is required for prostate bud induction. *Differentiation*.

[210] Hour MJ, Tsai SC, Wu HC (2012). Antitumor effects of the novel quinazolinone MJ-33: inhibition of metastasis through the MAPK, AKT, NF-kappaB and AP-1 signaling pathways in DU145 human prostate cancer cells. *International Journal of Oncology*.

[211] Shimizu T, Tolcher AW, Papadopoulos KP (2012). The clinical effect of the dual-targeting strategy involving PI3K/AKT/mTOR and RAS/MEK/ERK pathways in patients with advanced cancer. *Clinical Cancer Research*.

[212] Blobe GC, Schiemann WP, Lodish HF (2000). Role of transforming growth factor *β* in human disease. *New England Journal of Medicine*.

[213] Massagué J, Blain SW, Lo RS (2000). TGF*β* signaling in growth control, cancer, and heritable disorders. *Cell*.

[214] Schmierer B, Hill CS (2007). TGF*β*-SMAD signal transduction: molecular specificity and functional flexibility. *Nature Reviews Molecular Cell Biology*.

[215] Massague J (2008). TGFbeta in cancer. *Cell*.

[216] Shi Y, Massagué J (2003). Mechanisms of TGF-*β* signaling from cell membrane to the nucleus. *Cell*.

[217] Miles FL, Tung NS, Aguiar AA, Kurtoglu S, Sikes RA (2012). Increased TGF-beta1-mediated suppression of growth and motility in castrate-resistant prostate cancer cells is consistent with Smad2/3 signaling. *Prostate*.

[218] Donkor MK, Sarkar A, Li MO (2012). Tgf-beta1 produced by activated CD4^+^ T cells antagonizes T cell surveillance of tumor development. *Oncoimmunology*.

[219] Wan X, Li ZG, Yingling JM (2012). Effect of transforming growth factor beta (TGF-beta) receptor I kinase inhibitor on prostate cancer bone growth. *Bone*.

[220] Wikstrom P, Stattin P, Franck-Lissbrant I, Damber JE, Bergh A (1998). Transforming growth factor beta1 is associated with angiogenesis, metastasis, and poor clinical outcome in prostate cancer. *Prostate*.

[221] Adler HL, McCurdy MA, Kattan MW, Timme TL, Scardino PT, Thompson TC (1999). Elevated levels of circulating interleukin-6 and transforming growth factor-*β*1 in patients with metastatic prostatic carcinoma. *Journal of Urology*.

[222] Shariat SF, Shalev M, Menesses-Diaz A (2001). Preoperative plasma levels of transforming growth factor beta1 (TGF-*β*1 strongly predict progression in patients undergoing radical prostatectomy. *Journal of Clinical Oncology*.

[223] Derynck R, Akhurst RJ, Balmain A (2001). TGF-*β* signaling in tumor suppression and cancer progression. *Nature Genetics*.

[224] Wakefield LM, Roberts AB (2002). TGF-*β* signaling: positive and negative effects on tumorigenesis. *Current Opinion in Genetics and Development*.

[225] Yang J, Wahdan-Alaswad R, Danielpour D (2009). Critical role of smad2 in tumor suppression and transforming growth factor-*β*-Lnduced apoptosis of prostate epithelial cells. *Cancer Research*.

[226] Ye L, Kynaston H, Jiang WG (2009). Bone morphogenetic protein-10 suppresses the growth and aggressiveness of prostate cancer cells through a Smad independent pathway. *Journal of Urology*.

[227] Vo BT, Khan SA (2011). Expression of nodal and nodal receptors in prostate stem cells and prostate cancer cells: autocrine effects on cell proliferation and migration. *Prostate*.

[228] Kang HY, Huang HY, Hsieh CY (2009). Activin A enhances prostate cancer cell migration through activation of androgen receptor and is overexpressed in metastatic prostate cancer. *Journal of Bone and Mineral Research*.

[229] Whyte JL, Smith AA, Helms JA (2012). Wnt signaling and injury repair. *Cold Spring Harbor Perspectives in Biology*.

[230] Angers S, Moon RT (2009). Proximal events in Wnt signal transduction. *Nature Reviews Molecular Cell Biology*.

[231] Thiele S, Rauner M, Goettsch C (2011). Expression profile of WNT molecules in prostate cancer and its regulation by aminobisphosphonates. *Journal of Cellular Biochemistry*.

[232] Lu W, Tinsley HN, Keeton A, Qu Z, Piazza GA, Li Y (2009). Suppression of Wnt/*β*-catenin signaling inhibits prostate cancer cell proliferation. *European Journal of Pharmacology*.

[233] Hall CL, Kang S, MacDougald OA, Keller ET (2006). Role of Wnts in prostate cancer bone metastases. *Journal of Cellular Biochemistry*.

[234] Kimelman D, Xu W (2006). *β*-Catenin destruction complex: insights and questions from a structural perspective. *Oncogene*.

[235] Huelsken J, Behrens J (2002). The Wnt signalling pathway. *Journal of Cell Science*.

[236] Whitaker HC, Girling J, Warren AY, Leung H, Mills IG, Neal DE (2008). Alterations in *β*-catenin expression and localization in prostate cancer. *Prostate*.

[237] Majid S, Saini S, Dahiya R (2012). Wnt signaling pathways in urological cancers: past decades and still growing. *Molecular Cancer*.

[238] Barolo S (2006). Transgenic Wnt/TCF pathway reporters: all you need is Lef?. *Oncogene*.

[239] Mosimann C, Hausmann G, Basler K (2009). *β*-Catenin hits chromatin: regulation of Wnt target gene activation. *Nature Reviews Molecular Cell Biology*.

[240] Zhao J, Yue W, Zhu MJ, Sreejayan N, Du M (2010). AMP-activated protein kinase (AMPK) cross-talks with canonical Wnt signaling via phosphorylation of beta-catenin at Ser 552. *Biochemical and Biophysical Research Communications*.

[241] Gullick WJ (1991). Prevalence of aberrant expression of the epidermal growth factor receptor in human cancers. *British Medical Bulletin*.

[242] Guturi KK, Mandal T, Chatterjee A (2012). Mechanism of beta-catenin-mediated transcriptional regulation of epidermal growth factor receptor expression in glycogen synthase kinase 3 beta-inactivated prostate cancer cells. *Journal of Biological Chemistry*.

[243] Marker PC (2008). Does prostate cancer co-opt the developmental program?. *Differentiation*.

[244] Fearnhead NS, Britton MP, Bodmer WF (2001). The ABC of APC. *Human Molecular Genetics*.

[245] Chesire DR, Ewing CM, Sauvageot J, Bova GS, Isaacs WB (2000). Detection and analysis of beta-catenin mutations in prostate cancer. *Prostate*.

[246] Richiardi L, Fiano V, Vizzini L (2009). Promoter methylation in APC, RUNX3, and GSTP1 and mortality in prostate cancer patients. *Journal of Clinical Oncology*.

[247] Pascal LE, Vêncio RZN, Page LS (2009). Gene expression relationship between prostate cancer cells of Gleason 3, 4 and normal epithelial cells as revealed by cell type-specific transcriptomes. *BMC Cancer*.

[248] Koh CM, Bieberich CJ, Dang CV, Nelson WG, Yegnasubramanian S, De Marzo AM (2010). MYC and prostate cancer. *Genes and Cancer*.

[249] Alao JP (2007). The regulation of cyclin D1 degradation: roles in cancer development and the potential for therapeutic invention. *Molecular Cancer*.

[250] Grigoryan T, Wend P, Klaus A, Birchmeier W (2008). Deciphering the function of canonical Wnt signals in development and disease: conditional loss- and gain-of-function mutations of *β*-catenin in mice. *Genes and Development*.

[251] Hall CL, Bafico A, Dai J, Aaronson SA, Keller ET (2005). Prostate cancer cells promote osteoblastic bone metastases through Wnts. *Cancer Research*.

[252] Bruxvoort KJ, Charbonneau HM, Giambernardi TA (2007). Inactivation of Apc in the mouse prostate causes prostate carcinoma. *Cancer Research*.

[253] Uysal-Onganer P, Kawano Y, Caro M (2010). Wnt-11 promotes neuroendocrine-like differentiation, survival and migration of prostate cancer cells. *Molecular Cancer*.

[254] Gupta S, Iljin K, Sara H (2010). FZD4 as a mediator of ERG oncogene-induced WNT signaling and epithelial-to-mesenchymal transition in human prostate cancer cells. *Cancer Research*.

[255] Liss MA, Schlicht M, Kahler A (2010). Characterization of soy-based changes in Wnt-frizzled signaling in prostate cancer. *Cancer Genomics and Proteomics*.

[256] Chesire DR, Isaacs WB (2003). *β*-Catenin signaling in prostate cancer: an early perspective. *Endocrine-Related Cancer*.

[257] He B, You L, Uematsu K (2004). A monoclonal antibody against Wnt-1 induces apoptosis in human cancer cells. *Neoplasia*.

[258] Yardy GW, Brewster SF (2005). Wnt signalling and prostate cancer. *Prostate Cancer and Prostatic Diseases*.

[259] Le Guellec S, Soubeyran I, Rochaix P (2012). CTNNB1 mutation analysis is a useful tool for the diagnosis of desmoid tumors: a study of 260 desmoid tumors and 191 potential morphologic mimics. *Modern Pathology*.

[260] Willert K, Brown JD, Danenberg E (2003). Wnt proteins are lipid-modified and can act as stem cell growth factors. *Nature*.

[261] Sharma M, Chuang WW, Sun Z (2002). Phosphatidylinositol 3-kinase/Akt stimulates androgen pathway through GSK3*β* inhibition and nuclear *β*-catenin accumulation. *Journal of Biological Chemistry*.

[262] Polakis P (2007). The many ways of Wnt in cancer. *Current Opinion in Genetics and Development*.

[263] Wend P, Holland JD, Ziebold U, Birchmeier W (2010). Wnt signaling in stem and cancer stem cells. *Seminars in Cell and Developmental Biology*.

[264] Reya T, Morrison SJ, Clarke MF, Weissman IL (2001). Stem cells, cancer, and cancer stem cells. *Nature*.

[265] Castellone MD, Teramoto H, Williams BO, Druey KM, Gutkind JS (2005). Medicine: prostaglandin E2 promotes colon cancer cell growth through a Gs-axin-*β*-catenin signaling axis. *Science*.

[266] Lepourcelet M, Chen YNP, France DS (2004). Small-molecule antagonists of the oncogenic Tcf/*β*-catenin protein complex. *Cancer Cell*.

[267] Rey JP, Ellies DL (2010). Wnt modulators in the biotech pipeline. *Developmental Dynamics*.

